# Modular Virus Capsid Coatings for Biocatalytic DNA Origami Nanoreactors

**DOI:** 10.1021/acsnano.5c10734

**Published:** 2025-10-08

**Authors:** Iris Seitz, Donna McNeale, Frank Sainsbury, Veikko Linko, Mauri A. Kostiainen

**Affiliations:** † Department of Bioproducts and Biosystems, 174277 Aalto University, Aalto 00076, Finland; ‡ Centre for Cell Factories and Biopolymers, Institute for Biomedicine and Glycomics, 5723 Griffith University, Nathan, Brisbane, QLD 4111, Australia; § Institute of Technology, University of Tartu, Tartu 50411, Estonia; ∥ LIBER Center of Excellence, 174277 Aalto University, Aalto 00076, Finland

**Keywords:** DNA origami, virus capsid proteins, biocatalysis, antigen targeting, nanoreactor

## Abstract

Protein cages and custom DNA structures have emerged as self-assembling nanocompartments to sequester enzymes and mimic the compartmentalization of naturally occurring biocatalytic reactions. Protein cages excel in gating the interaction between enzyme and substrate, which can be affected by the physicochemical properties of protein units, whereas the high addressability of DNA origami allows stoichiometric control over the enzyme loading and precise positioning of enzymes. Nevertheless, both approaches would benefit from overcoming the challenges related to controlled enzyme loading and substrate flux, which could be resolved by combining the two nanomaterials. Here, we assemble virus capsid proteins on an enzyme-loaded DNA origami nanoreactor in a modular manner and demonstrate size-selective uptake of substrate molecules depending on the amount and type of capsid protein used for the encapsulation. The capsid cage also protects the biocatalytic unit from degradation, and further functionalization of the reactor surface with an antibody fragment allows targeting for delivery purposes. Thus, our approach provides an attractive platform not only for biomedical applications but also, because of its modularity, for rapid investigation of the physicochemical properties of capsid proteins.

## Graphical abstract

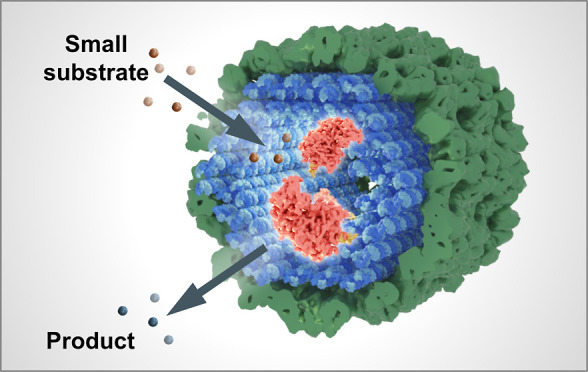

Micro- and nanoscale compartments are essential in living organisms, since they provide a unique environment to promote chemical reactions with high efficiency and specificity by ensuring high enzyme and substrate concentrations while segregating competing cross-reactions and protecting possible intermediates.^1^ These intriguing features found, for instance, in peroxisomes in eukaryotes or carboxysomes and encapsulins in prokaryotes,^2–4^ have inspired the development of artificial nanocontainers^5^ based on liposomes or polymersomes. However, poor retention efficiency and low permeability limit their application.^6^ Protein cages,^7–9^ and in particular virus-like particles (VLPs), which echo native viruses with respect to biocompatibility, stability, and uniformity of particle size without being infectious, offer an attractive alternative due to their outstanding self-assembly properties in vitro.^10–13^ The highly ordered protein capsid results in the protection of encapsulated enzymes from proteases or denaturation,^14,15^ while simultaneously allowing control over substrate flux by providing defined capsid pore size and surface charge.^16,17^ Despite a variety of encapsulation strategies, including nonspecific, noncovalent, and covalent approaches, stoichiometric control over the cargo remains challenging, which in return can negatively impact catalytic activity due to overcrowding or steric hindrance.^18^

A high degree of control over enzyme type, amount and precise location can be achieved by using fully addressable DNA origami structures.^19^ The DNA origami technique,^20^ in which a long, single-stranded DNA (ssDNA) scaffold is folded into well-defined two- or three-dimensional structures by hybridization with short, ssDNA oligonucleotides,^20–22^ provides programmable structures especially suitable for templating enzymatic cascade reactions.^23^ Enzymatic reactions on DNA origami structures have been successfully performed using robust and commercially available enzymes like horseradish peroxidase (HRP), but also more biocatalytically relevant processes including protein unfolding and degradation have been demonstrated.^24^ Interactions between enzymes and substrates can be controlled with nanopore systems,^24^ and externally triggered conformational changes have also been implemented in enzyme-encapsulating nanocages,^25,26^ however, container walls made of a double-layer of DNA helices have been suggested to be permeable to small cargo molecules.^26,27^

In this article, we explore the synergy between virus capsid-based protein cages and DNA origami for biocatalysis to gain control over enzyme–substrate interactions while regulating the cargo load. To this end, we exploit a hollow, tubular DNA origami nanoreactor (NR) structure to house the enzyme in a spatially confined environment. Owing to the phosphate groups in the DNA backbone, DNA origami structures are negatively charged. The outer surface of the DNA origami NR can therefore serve as a platform for the assembly of virus capsid proteins (CPs), resulting in a well-defined protein shell, which in return can control the flux of substrate molecules to the enzyme site (Figure 1a). The permeability of the capsid was investigated using three enzyme substrates that differ in molecular weight. By adjusting the stoichiometric ratio between the CPs and the DNA origami, size selectivity toward the substrate molecules was observed. In addition, fluorescently labeled DNA strands were used to study the addressability and accessibility of ssDNA overhangs protruding from the DNA origami NR in the presence of different levels of CP coatings. Further, we also showed that the nanoreactor could be immobilized by functionalizing its surface with antigen-targeting moieties.

**Figure 1:**
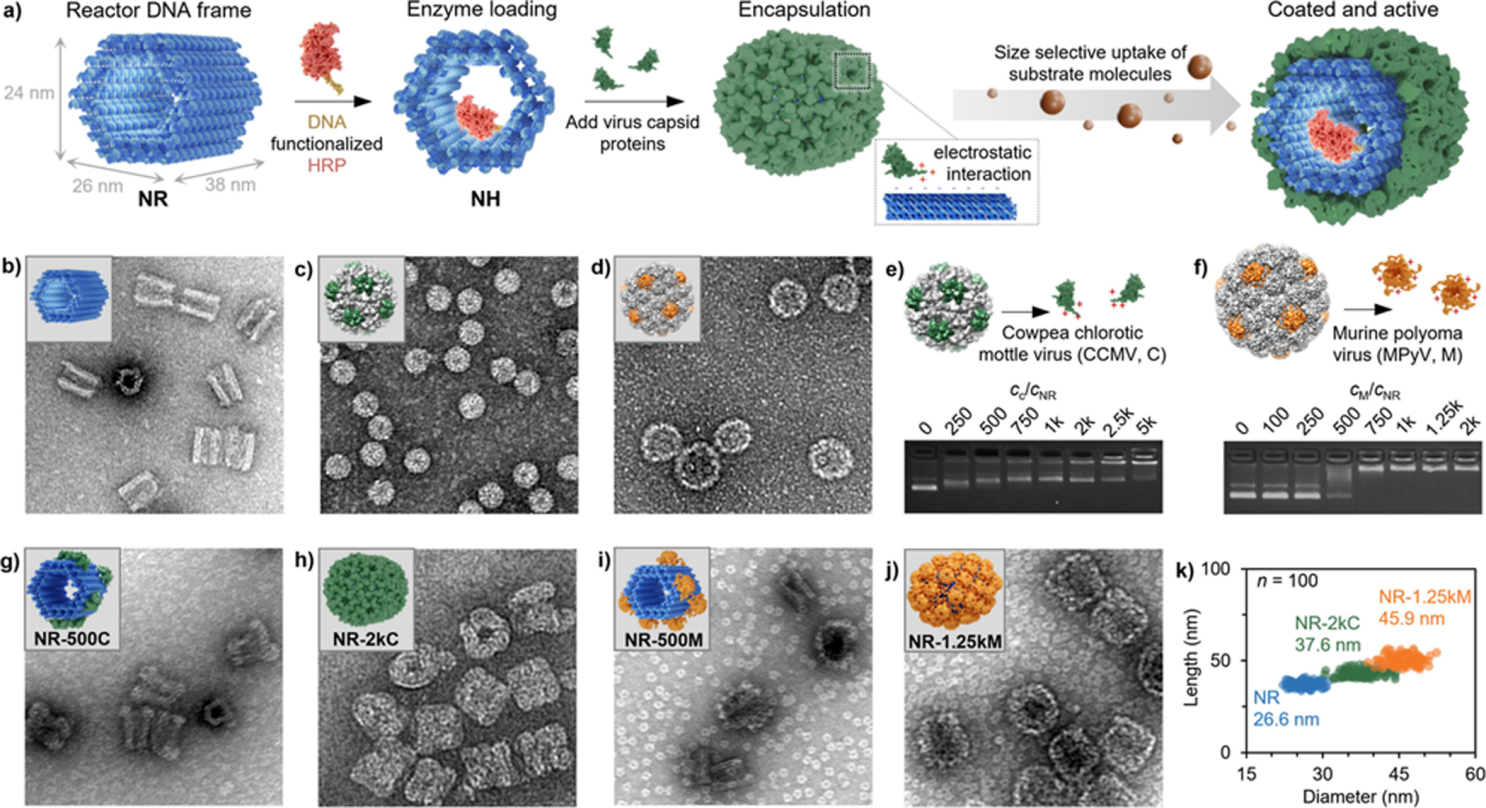
Structural characterization of the virus-encapsulated DNA origami NR. (a) The DNA origami nanoreactor (NR) frame was loaded with DNA-functionalized HRP to obtain the HRP-loaded NR (NH) catalytic unit. The addition of different types of CPs (CCMV in green, MPyV in orange) resulted in encapsulation of the DNA origami due to the electrostatic interactions between the negatively charged phosphate backbone of the DNA and positive amino acid residues at the proteins’ *N*-termini. (b) Negative-stain TEM image (image width corresponds to 250 nm) of NR. (c,d) Negative-stain TEM images (image width corresponds to 250 nm) of native CCMV (c) and MPyV VLPs (d). (e,f) Agarose gels monitoring the shift in electrophoretic mobility when mixing NR with increasing concentration of CCMV CPs (e) and MPyV CPs (f). (g–j) Negative-stain TEM images (image width corresponds to 250 nm) showing the partial and complete encapsulation of the DNA origami, represented by NR-500C (g), NR-2kC (h), NR-500M (i), and NR-1.25kM (j). (k) Statistical analysis of the size distribution of the encapsulated NR measured from TEM. The values are given as an average of *n* = 100 particles.

## Results

### Design and Assembly of Virus-Encapsulated DNA Origami Nanoreactors

We started by designing and characterizing the DNA origami NR frame which serves as the centerpiece for the stepwise assembly of the virus capsid-coated reactor (Figures 1a,b and S1). The DNA double helices in the NR frame are arranged along a honeycomb lattice, resulting in the NR resembling the shape of a hexagonal prism with outer dimensions corresponding to ca. 26 nm × 24 nm × 38 nm (*w* × *h* × *l*). To prevent dimerization, the edges of each double helix of the NR were passivated with 8-nucleotide (nt) long ssDNA poly-T overhangs. Twenty-six of these overhangs are exchangeable (NR-E) to enable the attachment of ATTO488 fluorophore (A488)-labeled oligonucleotides (17 nt), additionally, 18 possible attachment sites are located along each face (NR-F). In order to ensure rigidity of the hollow NR (channel diameter (*d*) of ca. 15 nm, similar to previously reported nanoreactor cavities^26,28,29^ ), the walls were made of two layers of DNA double helices. Four ssDNA overhangs (16 nt) protrude from the inner surface of the NR to facilitate enzyme loading. Here, we used the well-characterized HRP as a model enzyme. For the loading process, HRP was first conjugated to an ssDNA oligonucleotide (13 nt) which is complementary to the ssDNA overhangs protruding from the interior of the NR. Then, the DNA-functionalized HRP was immobilized to the NR via hybridization, thus forming an HRP-loaded NR (NH) catalytic unit.

Subsequently, the DNA origami NR (with or without the enzymatic payload) is complexed with CPs of either cowpea chlorotic mottle virus (CCMV, Figures 1c and S2a, diameter *d* = 25.8 ± 0.9 nm, given as average (avg.) ± s.d. throughout) or murine polyomavirus (MPyV; assembled VLPs shown in Figures 1d and S2b, *d* = 42.0 ± 3.0 nm). CCMV is known to adopt a quasi-icosahedral *T* = 3 symmetry by arranging 180 CP copies into 20 hexamers and 12 pentamers,^30^ while MPyV CPs assemble entirely into pentamers, resulting in a pseudo *T* = 7d symmetry.^31^ Both CCMV and MPyV CPs are known for their polymorphic behavior upon reassembly^32–37^ which can also be templated by organic and inorganic materials;^38–44^ they have previously shown their capability to assemble on rod-like shaped DNA origami with varying diameters.^44^ In these examples, the CP assembly process is driven by protein–protein and electrostatic interactions: the DNA origami is negatively charged due to its phosphate backbone and the *N*-termini of the CPs, on the other hand, are rich in positively charged amino acid residues.^45–47^ The DNA origami thereby guides the CPs into the desired shape, similar to long single-stranded RNA, which has been reported to promote the in vitro assembly of CPs into icosahedral particles.^48^

The complexation reaction between NR and CPs, either CCMV CPs, denoted as C, or MPyV CPs, denoted as M, was performed with an excess of CPs. The excess is defined as the molar ratio between CPs and NR, and is indicated by *c*_CP_/*c*_NR_, i.e., an excess of 500 corresponds to 500 CP monomers mixed per one NR. The formation of the complexes was monitored on the basis of their shift in electrophoretic mobility using agarose gel electrophoresis (AGE). When complexed with CCMV CPs (Figure 1e), the mobility of the NR was observed to gradually decrease with increasing excess of CPs, until it reached a first plateau at an excess of 750. The use of excesses greater than 2k (k denoting the multiplier 1000) led to an intensity decrease of the leading band. Simultaneously, a band with reduced mobility appeared, suggesting the nucleation of a second CP layer. Multilayer formation has previously been observed for rod-like assemblies and is caused by the interaction between free CPs and negatively charged patches on the outer surface of the CPs already assembled on DNA origami.^44^

A decrease in the intensity of the NR leading band was also observed upon the addition of MPyV CPs (Figure 1f), with the most prominent shift occurring at an excess of 500. A plateau was reached at an excess of 750, after which the mobility remained unchanged despite increasing CP concentration.

To get a comprehensive understanding of the impact of the protein shell on the enzyme activity, samples with low and high CP excesses were further examined, being 500 and 2k for CCMV CPs as well as 500 and 1.25k for MPyV CPs. For simplicity, the complexed samples are denoted as NR-YZ or NH-YZ, i.e., the nanoreactor without or with enzymatic payload is complexed with Y excess of the CP type Z. The smear observed for NR-500M in AGE suggests that the sample is heterogeneous with respect to how far the encapsulation had progressed. Similarly, NR-500C migrated faster than samples that plateaued, hinting that the protein shell is only partially developed. Negative-stain transmission electron microscopy (TEM) of NR-500C (Figures 1g and S3a) confirmed the interaction between NR and CPs. The CPs were preferentially bound around the edges, without fully covering the surface of the DNA origami. Increasing the excess to 2k (Figures 1h and S3b) resulted in an increase of the dimensions of the NR due to complete encapsulation. A similar behavior was observed for MPyV CPs. While only a few pentamers were bound to the NR surface at low excess (Figures 1i and S3c), an ordered protein shell was detected for NR-1.25kM (Figures 1j and S3d). The change in dimensions was most pronounced with regard to the width and height of the complexes. However, to simplify the measurements, the NR is considered as a cylinder with a circular base area instead of a hexagonal prism. Statistical analysis revealed an increase in the diameter from 26.6 ± 2.1 nm to 37.6 ± 3.1 nm for NR-2kC and 45.9 ± 2.6 nm for NR-1.25kM (Figure 1k).

### Surface Accessibility/Addressability of Capsid Protein-Coated DNA Origami

To evaluate the addressability of CP-coated DNA origami, we probed the availability of ssDNA overhangs (16 nt) protruding from the edges (NR-E) or along the surface (NR-F) (Figure 2a). To this end, the coated NR-variants were incubated overnight (ON) at ambient temperature in a solution containing the complementary A488-labeled DNA oligonucleotides. The success of the hybridization was monitored by AGE (Figures 2b–e and S4). Ethidium bromide (EtBr) was used for the detection of nucleic acids whereas imaging under blue light, Alexa488 (Al488) channel, allowed the visualization of the A488 fluorophore. To facilitate detection after the possible hybridization, the NR-CP complexes were incubated with heparin (Supporting Information Note S4). The polyanion served as a competing agent, which triggered the disassembly of the complexes and therefore increased the electrophoretic mobility. NR-variants incubated without A488-labeled oligonucleotides (first lane from left) and the uncoated NR-variants (second lane from left) were used as controls throughout. When comparing the controls to each other after annealing, a slight shift in the mobility was observed in the EtBr channel, which suggests successful hybridization of the A488-labeled oligonucleotides to the overhangs (Figure 2b,d, top, respectively). Note that the buffer environment differs between CCMV CP and MPyV CP-coated samples (see Experimental Section "Complexation of Nanoreactor with Virus Capsid Proteins"). The signals emerging in the Al488 channel (bottom) additionally confirmed the hybridization of A488-labeled strands to the NR variants. The control samples were found to show a similar behavior regardless of whether the ssDNA overhangs were positioned on the edges (left) or the surface (right).

**Figure 2:**
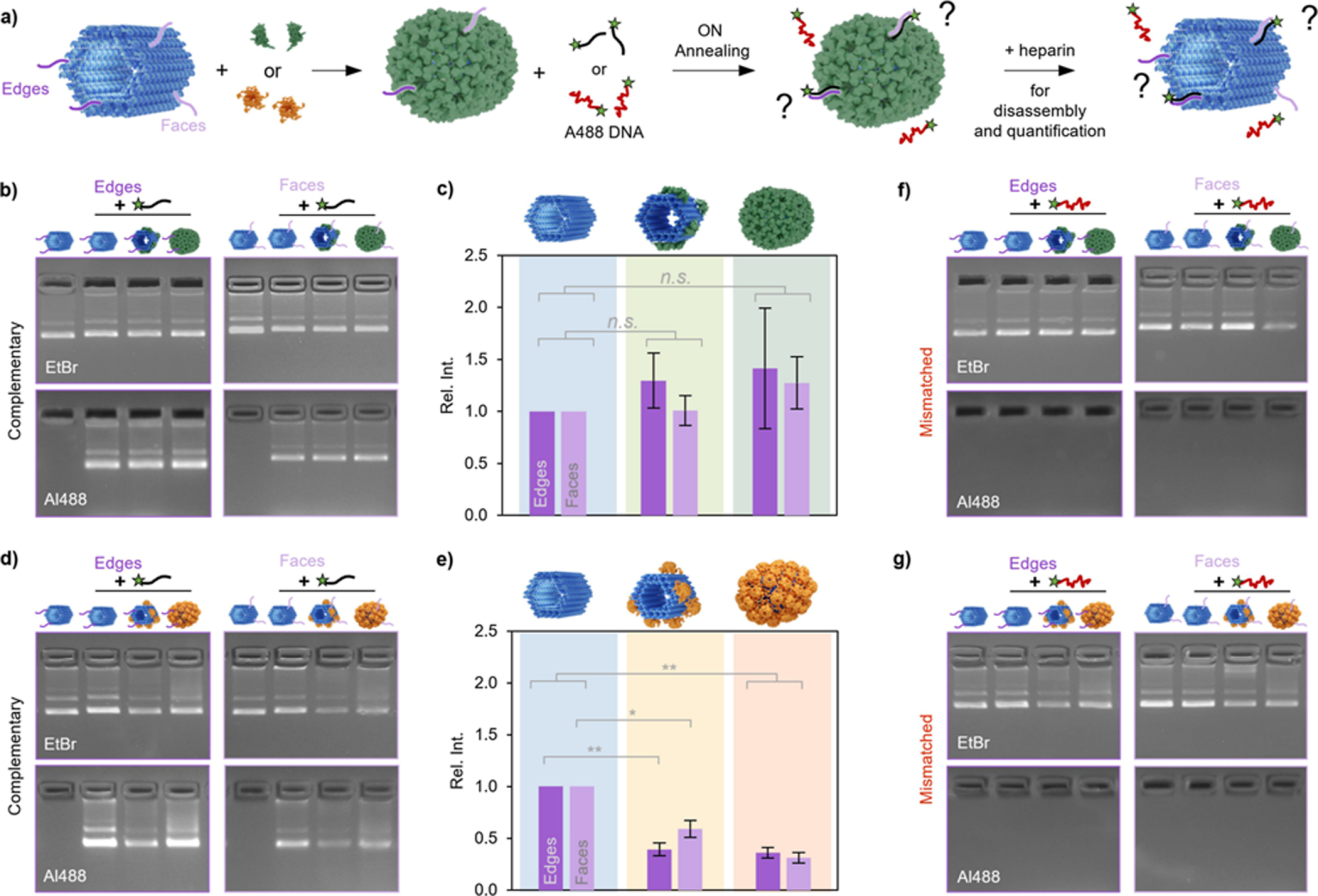
Investigating the surface accessibility/addressability of CP-coated DNA origami. (a) The variants NR-E (purple edge ssDNA overhangs) and NR-F (light purple face ssDNA overhangs) were complexed with CCMV or MPyV CPs, followed by an overnight (ON) incubation with A488-labeled oligonucleotides, which were either complementary (black) or have mismatched sequence (red). To determine the accessibility of the ssDNA overhangs, the complexes were disassembled with heparin just before detection. (b) Agarose gels imaged under UV (EtBr channel, top) and blue light (Al488 channel, bottom) were used to assess the accessibility for NR-E (left) and NR-F (right) when complexed with CCMV CPs. (c) Quantification of the fluorescence intensities obtained from (b) showed that CCMV CPs do not restrict the access for short oligonucleotides. (d) Agarose gel to determine the accessibility to NR variants complexed with MPyV CPs and (e) quantification of the obtained fluorescence intensities. (f,–g) To exclude unspecific interactions causing the fluorescence signal, an A488-labeled oligonucleotide with mismatched sequence was incubated with NR-variants complexed with CCMV CPs (f) or MPyV CPs (g). The values are given as avg. ± s.d. of three independent replicates with *, **, and n.s. denoting the significance (*P* < 0.05, *P* < 0.01, and not significant).

When samples complexed with CCMV CPs were incubated with the A488-labeled oligonucleotides, no apparent change in the intensity of the bands could be detected. For a more accurate comparison, the A488 fluorescence intensities of each lane were quantitatively estimated and compared to the respective A488-functionalized control sample (Figure 2c). Our results indicate the accessibility to surface decorations after encapsulation of the DNA origami structure with CCMV CPs, a property which has also been reported for oligolysine-PEG coatings.^49^ On the other hand, a significant decrease in the fluorescence intensity was detected for structures complexed with MPyV CPs (Figure 2d,e). Interestingly, the faces of NR-500M appeared to be slightly more accessible than its edges. This could be an indication that the coating preferentially develops from nucleation sites along the edges, similar to the behavior observed for CCMV CPs (Figure 1g). However, it should be noted that the preferential position of nucleation sites is structure-specific and dependent on the dimensions of the template.^44,50,51^

The differences in accessibility could be assigned to the physicochemical properties of the CPs. In solution, free CCMV CPs are dimeric units, whereas free MPyV CPs are pentameric capsomers. CCMV is known for its swelling behavior when the pH is increased from acidic to neutral.^30^ Thereby, the pore size of the virus was found to increase by ca. 5 Å, from 8.1–10.6 Å to 13.3–17.4 Å.^30^ The swelling triggers the exposure of patches of negative electrostatic potential which are located at the capsid shell openings.^52^ The inner capsid surface retains the positive charge of the *N*-termini, however, patches of negative electrostatic potential are also exposed.^52^ Based on the buffer conditions during complexation (150 mM NaCl, pH ∼ 7.3) and cryo-reconstruction,^44^ the CPs are expected to adopt to the swollen conformation, i.e., the larger pores sizes. In contrast, the pore formed by the pentameric arrangement of MPyV CPs was calculated to have a diameter of 8.6 Å.^53^

To exclude that the fluorescence signal originates from unspecific interactions between CPs and A488-labeled oligonucleotides and possible incomplete release of the CPs despite heparin treatment, the experiments were repeated using an A488-labeled DNA oligonucleotide with a mismatched sequence (sequence not complementary to ssDNA overhangs) (Figure 2f,g). However, neither a decrease in the electrophoretic mobility compared to NR-variants incubated without the A488-labeled strands, nor a fluorescence signal could be detected, confirming the specificity of our approach.

### Biocatalytic Activity of NH

After establishing the protein shell of our system, the catalytic unit NH was characterized. We selected HRP as the model enzyme due to its robust nature and variety of substrates, among others, *o*-phenylenediamine (oPD), 3,3′,5,5′-tetramethylbenzidine (TMB) and 2,2′-azino-bis­(3-ethylbenzothiazoline-6-sulfonic acid) (ABTS), which allow colorimetric readout assays.^54^ Since the detection of single HRP proteins is not reliable in TEM, the accessibility of the enzyme-loading sites located at the inner surface of the reactor was first confirmed using 5 nm diameter gold nanoparticles (AuNPs) (Supporting Information Note S5). To this end, the surface of the AuNPs was functionalized with DNA oligonucleotides. The oligonucleotide sequence is complementary to the four ssDNA overhangs protruding from the inner surface of the NR that facilitate enzyme loading, i.e., the AuNPs are loaded in the same manner as the enzyme. TEM revealed AuNPs in the NR cavity, thus confirming the accessibility of each of the four enzyme loading sites (Figure S6). However, the highest yield of one AuNP per NR was achieved in the presence of all four loading sites (Figure S7).

The catalytic unit NH was obtained by annealing the DNA-functionalized HRP to the DNA origami NR. Since the DNA-functionalized HRP was used in excess, unbound enzymes were removed using PEG precipitation^55^ before adding the substrate (Supporting Information Note S6). Compared to free DNA-functionalized HRP in the presence of DNA origami (Figure 3a, red), the confinement of HRP in the DNA origami NR (Figure 3a, blue) resulted in an approximate 5-fold decrease in ν_max_ (Figure 3b) when oxidizing the standard substrate oPD in the presence of H_2_O_2_. In contrast, no loss in *K*_M_ was observed. A possible explanation for this could be that while the free HRP was kept at a concentration of 2 nM, the exact enzyme concentration in the NH sample is unknown. In the design, there are four protruding attachment strands along the inner reactor surface in a circular arrangement, resulting in a theoretical maximum HRP concentration of 8 nM when assuming a loading yield of 100%, as the DNA origami concentration was kept constant at 2 nM for all experiments. However, loading of more than one enzyme per reactor seems highly unlikely due to steric hindrance, and thus, the corresponding upper limit for the final enzyme concentration would likely be in the range of ∼2 nM. To further explore the HRP loading efficiency, the activity of NH-variants with only one ssDNA oligonucleotide overhang was recorded (Figure S8). While ν_max_ decreased by approximately 3-fold in comparison to NH (four ssDNA oligonucleotide overhangs), *K*_M_ and *k*_cat_ remained, as expected, unchanged, as ν_max_ is dependent on the enzyme concentration.

**Figure 3:**
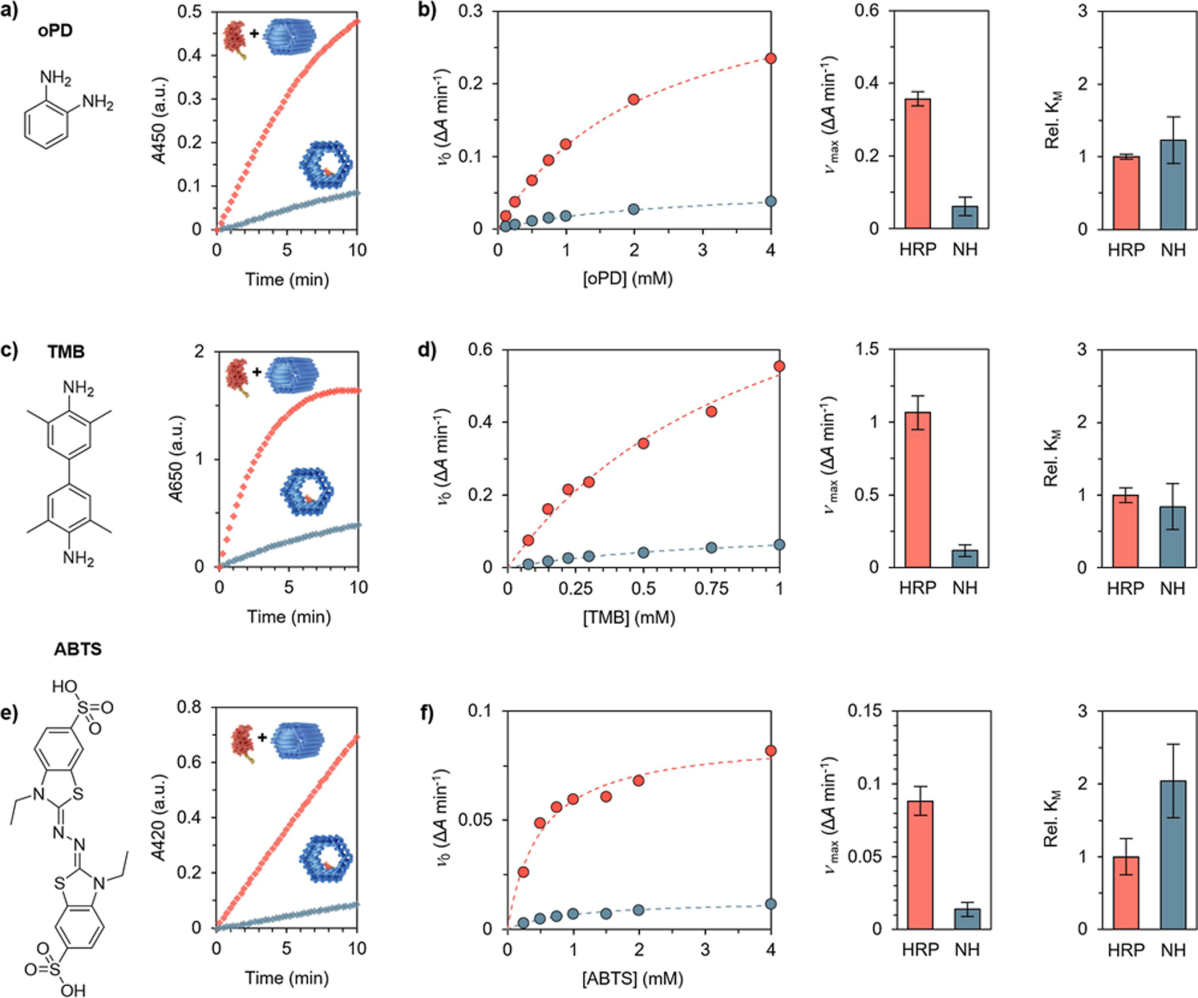
Biocatalytic characterization of NH. (a) Absorbance over time for monitoring the oxidation of oPD (0.5 mM) in the presence of HRP and H_2_O_2_. The NH (blue) was compared to free DNA-functionalized HRP (red). (b) Analysis of the product formation by applying the Michaelis–Menten model, visualized as the initial rate vs substrate concentration-plot (left) from which ν_max_ (middle) and *K*_M_ (right) were determined. *K*_M_ is given relative to free DNA-functionalized HRP. (c) Absorbance over time monitoring the product formation using TMB (0.75 mM) as substrate and (d) analysis of the catalytic activity. (e,f) The kinetics for ABTS (2 mM) were determined based on the absorbance at 420 nm. The measurements were performed as three independent replicates and the values are given as avg ± s.d.

The observed decrease of the reaction rate of NH compared to unbound HRP could also be attributed to the immobilization and spatial confinement of HRP, i.e., both substrate molecules need to diffuse into the reactor. Structural changes in the enzyme, inhibition of substrate diffusion, and microenvironment effects have been shown to impact enzymatic activity. Work by O’Brien et al.^56^ demonstrated that three of the six lysine residues in HRP are surface exposed and amenable to modification. Although these lysine residues, Lys174, Lys232, and Lys241, are not directly involved in the active site, molecular dynamic simulations indicated that modification of these residues results in structural changes that impact active site accessibility.^57^ Ergo, covalent conjugation of DNA oligonucleotides, particularly to Lys174 which is located near the active site, and further hybridization to the NR, could restrict substrate access. However, since the *K*_M_ values for unbound HRP and NH are similar, this is likely not the case. Similarly, a previous study on the immobilization of HRP on polymer brushes via covalent conjugation through lysine residues concluded that the 100-fold decrease in activity of the immobilized HRP compared with free HRP was not attributable to blockage of the active site from conjugation.^58^ Another factor that may influence the activity of NH is the environment of water molecules near the DNA origami surface. The highly negatively charged environment originating from the phosphate backbone of the DNA origami causes a high-density hydration layer to form. Substrate concentration within the hydration layer, and therefore near the immobilized HRP, will differ to that near the free HRP depending on the hydration free energy of the substrate.^59^ Consequently, hydrophobic substrates will be diluted within the hydration layer and will lead to reduced catalytic rates. The largest decrease in ν_max_ was observed for TMB, which simultaneously is the most hydrophobic molecule of the substrates used. Furthermore, a pH gradient might be established, resulting in a lower local pH at the surface of the DNA origami than in the bulk solution.^60^ Given that HRP is stable within the pH range of 5–9, with the optimum pH at pH 6–8,^61^ it is possible that the pH at the DNA origami surface drops below this range, creating a less favorable environment and subsequently reducing the catalytic rate of HRP.

To further investigate the behavior of NH, larger substrate molecules, TMB (Figure 3c,d) and ABTS (Figure 3e,f) were selected. Like oPD, the reaction rates for these two substrates decreased drastically in comparison to unbound HRP, while the changes in *K*_M_ were moderate.

### Controlling the Substrate Influx

With the NH unit characterized, showing that the DNA (origami) environment clearly impacts the catalytic activity, the ability to modulate the enzyme activity by applying a capsid coating was investigated. To this end, the different CP-coated NHs were incubated with the standard substrates oPD, TMB, and ABTS, and their oxidation products were monitored over time (Figures 4a–c and S9–S12), from which ν_max_ (Figure 4d–f, middle) and *K*_M_ (Figure 4d–f, bottom) were determined. While the performance of NH (blue) oxidizing oPD is barely affected by the addition of a moderate amount of CCMV CPs (Figure 4a,d, light green), an approximate 2-fold decrease in ν_max_ was observed for the fully encapsulated reactor (NH-2kC, Figure 4a,d, green). Similarly, a decrease in ν_max_ was detected with NH-500M (Figure 4a,d, yellow) and NH-1.25kM (Figure 4a,d, orange), suggesting the possibility to tune the enzyme–substrate interaction by carefully choosing the amount and type of CP (Figures S13 and S14). Intriguingly, the impact of the CP coating on gating the substrate becomes more pronounced when changing the substrate molecule. In the presence of TMB (Figure 4b,e), the product formation in NH-1.25kM (Figure 4b,e, orange, Figure S9a) was negligible, making an accurate determination of the kinetic parameters impossible. A similar behavior was also observed for NH-500M in the presence of ABTS (Figure 4c,f, yellow, Figure S9b), indicating that the CP coating restricts the substrate from entering the reactor. In addition, *K*_M_ was considerably increased for NH-2kC (Figure 4c,f, dark green) suggesting a lower affinity of the ABTS to HRP or a decrease in availability. In comparison, NH-500C (light green) and NH-2kC (dark green) show similar *K*_M_ values for both oPD and TMB as substrates, implying restricted access of ABTS to the enzyme as main reason. Limited accessibility is not exclusive for protein encapsulation as it has also been observed with polymer coatings.^62^

**Figure 4:**
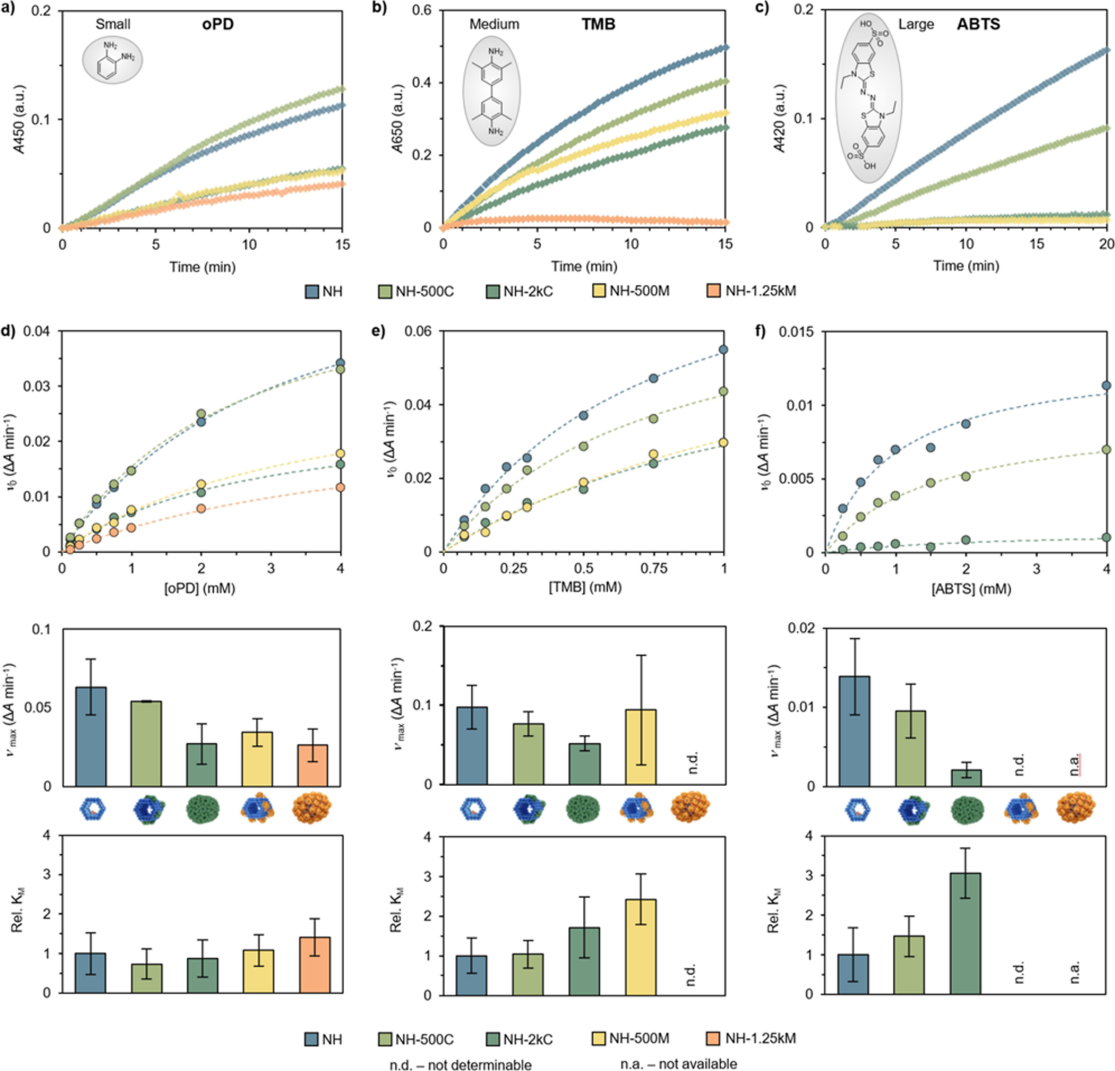
Investigating the effect of the CP coating on the catalytic activity. (a–c) The absorbance was recorded over time to follow product formation using oPD (a, 0.5 mM), TMB (b, 0.75 mM) and ABTS (c, 2 mM) as substrates for NH (blue), NH-500C (light green), NH-2kC (green), NH-500M (yellow), and NH-1.25kM (orange). (d–f) The kinetic parameters of the enzymatic reaction were determined by applying a Michaelis–Menten model and are presented by plotting the initial rate vs substrate concentration (top), a comparison of ν_max_ (middle) and *K*_M_ (bottom), which is given relative to NH (*K*_M_ of NH set to 1) (blue). The measurements were performed as three independent replicates and the values are given as avg. ± s.d.

The ability of the CPs to gate the interactions between enzyme and substrate is assigned to their physicochemical properties, especially to the pores that are formed at the CP–CP interface when the CPs self-assemble. Size-dependent uptake has previously been shown with P22,^16^ and the results obtained from the catalysis experiments are in line with the observations from probing the accessibility of DNA origami surface with DNA oligonucleotides. Despite the similarity of the pore sizes between CCMV (8.1–10.6 Å) and MPyV (8.6 Å), MPyV-coated structures exhibited a greater decrease in catalytic activity when compared to CCMV-coated structures. The reported pore sizes are calculated on the basis of 3D reconstructions; however, the effective pore size can differ due to the steric impact of amino acid side chains and the electrostatic potential around the pore. Possible interactions between the substrate and amino acids located around the pore can influence permeability.^17,63^ These differences in the effective pore sizes of MPyV- and CCMV-coated NR are likely a contributing factor in the observed catalytic activities.

### Functionalization of the NH Surface with Targeting Moieties

For the development of DNA-based tools for biomedical applications, targeting has gained an increasing interest to ensure delivery to the desired site while decreasing potential side effects.^64^ To this end, DNA nanostructures have been functionalized with diverse targeting moieties, including antibodies, affibodies, ligands, and aptamers. Most of them are site-specifically attached to the DNA nanostructures, however, its negative surface charge can be also exploited to facilitate functionalization through electrostatic interactions.^65^

Here, as a proof-of-concept, the NR was complexed with a single-chain antibody-fragment (anti-HER2) against human epidermal growth factor receptor 2 (HER2). As demonstrated previously,^65^ anti-HER2 was conjugated to a positively charged synthetic binding domain (aH, Supporting Information Note S7) to facilitate electrostatic interactions with the DNA origami. For the complexation reaction, which was monitored by AGE (Figure 5a), no shift in electrophoretic mobility could be observed when aH was added, unless a large excess of 240× was employed. The retention of the complexes in the well suggests the formation of aggregates, a behavior that had previously been reported for small proteins carrying a synthetic domain, regardless of the shape of the DNA origami.^65,66^ The onset of aggregation could already be detected at an excess of 30×, while NRs complexed with 15× excess appeared mainly as discrete structures under TEM (Figures 5b and S16). Since aggregation would be unfavorable, the NR complexed with 15× excess (NH-aH) was chosen for further studies. To assess the targeting properties of NH-aH, a fluorescence-based plate immunoassay was performed (Figure 5c). Briefly, the well surface (96-well plate) was decorated with the extracellular domain (ECD) of HER2, and bovine serum albumine (BSA) was used as a blocking agent to reduce unspecific binding. Subsequently, NH-aH complexes, which were labeled with A488-labeled oligonucleotides by using the NR-E variant, were added into the well. The interaction between antibody and antigen resulted in the immobilization of NH on the surface, which was monitored by measuring the fluorescence intensity. The binding efficiency is given as the ratio of the fluorescence intensities measured for wells containing (+) and lacking (−) HER2 to account for any unspecific interaction (Figures 5d and S17). While NH (blue, sample 1) behaved similarly as the blank (dark blue, sample 6), and therefore, did not exhibit specific binding to the plate, a significant increase in the fluorescence intensity could be detected for NH-aH (red), suggesting successful targeting. The immobilization did not prohibit the nanoreactor (NH-aH) from its catalytic activity once oPD was added together with H_2_O_2_ (Figure 5e). For proof-of-principle, partially coated reactors were immobilized. There, the synthetic domain of aH can either bind to noncoated areas of the DNA origami or negatively charged patches located on the outer surface of CCMV CPs. Both NR-50C (sample 3) and NR-150C (sample 4) resulted in a net increase in fluorescence compared to NH (sample 1). However, an increase in unspecific binding was observed alongside the increase in CP coating (NR-500C, sample 5). Due to the complexity of the assay, the decrease in signal is likely caused by several factors. For one, the two binding domains, being either the positively charged amino acids on the *N*-terminus of the CP or the synthetic domain added to aH, most likely have different binding affinities to the DNA origami. The competitiveness for binding might lead to dissociation of aH due to the amount of CPs being significantly higher than for aH. A similar behavior has been reported for excessive amounts of BSA in the sample.^65^ Additionally, possible unspecific interactions between the CPs and BSA, which had been immobilized on the plate surface, could contribute. Nevertheless, the performance in a cellular environment might differ. To this end, treatment with DNase I (Figure 5d, inset, Supporting Information Note S8) demonstrated that even low amounts of CPs enhance the stability of the complexed NR (NR-150C, sample 4) when compared to plain NR (sample 1). Both NR and NR-150C displayed an onset of digestion at 2.5 KU mL^–1^. While NR is readily digested when increasing the DNase I concentration, NR-150C displayed a leading band upon treatment with up to 10 KU mL^–1^, though with higher mobility, suggesting partial digestion of uncoated areas.

**Figure 5:**
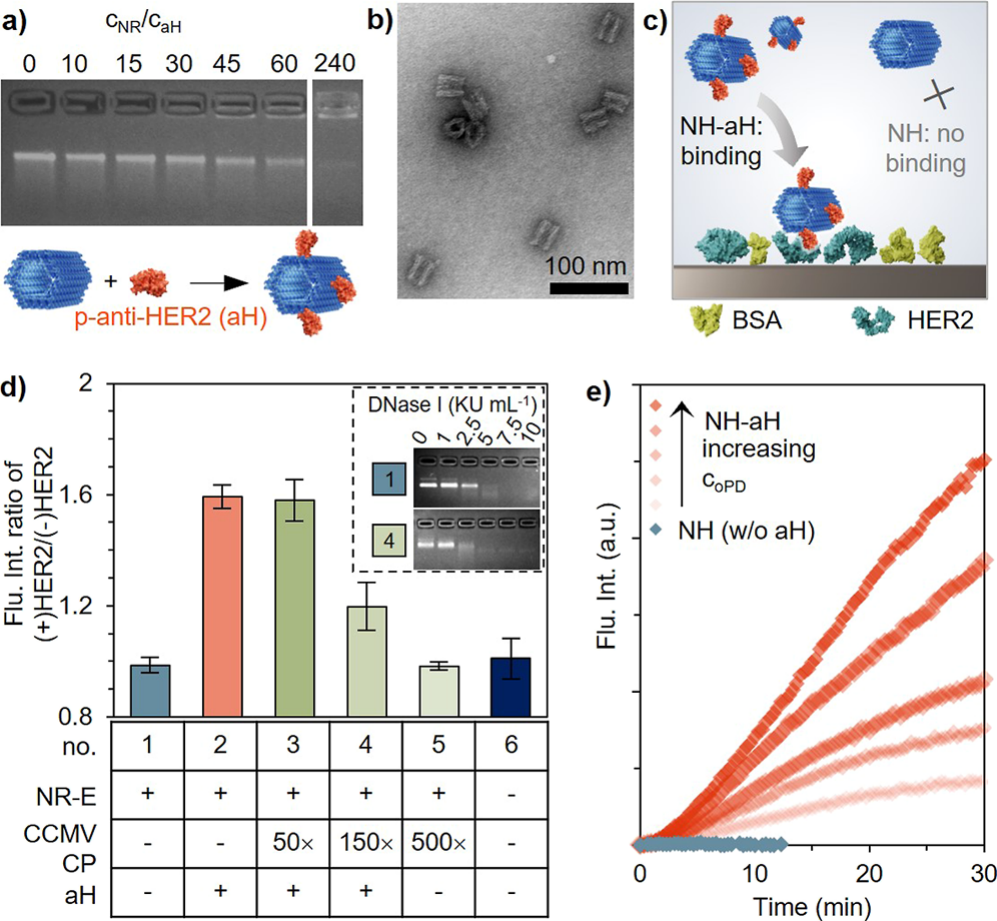
(a) Agarose gel electrophoresis was used to monitor the complexation between the NR and the targeting moiety anti-HER2. Anti-HER2 was conjugated to a synthetic binding domain (aH), resulting in the complexation being mediated by electrostatic interactions. (b) Negative-stain TEM image of NR-aH, an NR which was complexed with 15× excess of aH. (c) Schematics depicting the fluorescence-based plate immunoassay. HER2 (ECD) was immobilized on the plate surface, and BSA was used a blocking agent, and NH-aH variants were incubated. (d) Successful binding of the nanostructure is determined from the fluorescence intensity (A488), given as ratio of (+)­HER2/(−)­HER2 to account for unspecific interactions. (e) The catalytic activity of immobilized NH was determined from the fluorescent oxidation product of oPD in the presence of H_2_O_2_.

## Conclusions

In conclusion, we have developed a modular nanoreactor that exploits the synergy between DNA origami and virus CPs to increase the tunability of enzymatic reactions. The stepwise assembly of CPs allows control over not only the type but also the amount of CP used, defining the degree of encapsulation and the permeability of the protein shell. This, in return, gates the interactions between enzyme and substrate. Additionally, our approach demonstrated an enhancement of the DNA origami stability in a nuclease-rich environment even for partially coated structures, but also offers further functionalization with targeting moieties either through electrostatic interactions or genetic engineering of CPs.^67^ The combination of biocompatible CPs^68,69^ with the high addressability of the DNA origami could be harnessed for cascade reactions making use of a specific microenvironment, and the reactor system could be implemented in various fields ranging from metabolic engineering^28,70,71^ to high-throughput enzymatic platforms. Moreover, due to its modularity, our platform presents a simple yet rapid approach for investigating the microenvironment inside biohybrid nanoreactors as well as physicochemical properties of CPs.^72^

## Experimental Section

### Folding and Purification of the DNA Origami

The NR structure was designed on a honeycomb lattice using caDNAno^73^ (Supporting Information Note S9) and its three-dimensional shape was predicted with the CanDo software.^74,75^ The structure (p7560 scaffold purchased from Tilibit Nanosystems, staple strands from Integrated DNA Technologies, Tables S1–S3) was folded in a one-pot reaction by gradually decreasing the temperature using a Proflex 3 × 32-well PCR system (Thermo Fisher). Briefly, the scaffold (final concentration of 20 nM) was mixed with 10× excess of staple strands in a buffered environment (‘folding buffer (FOB)’) containing 1× Tris-acetate-EDTA (1 × TAE) supplemented with 15 mM MgCl_2_. The staple strands were annealed by cooling from 65 to 59 °C at a rate of −4.0 °C h^–1^ and from 59 to 40 °C at a rate of −0.33 °C h^–1^, followed by cooling to 20 °C until the program was manually stopped.

The folded DNA origami structures were purified by removing excess staple strands using polyethylene glycol (PEG) precipitation.^55^ First, the DNA origami solution was diluted to a concentration of ca. 5 nM using 1× FOB, followed by mixing with PEG precipitation buffer (1 × TAE, 15% (w/v) PEG8000, 505 mM NaCl) at 1:1 volume ratio, and a centrifugation step at 14,000*g* for 30 min at room temperature using an Eppendorf 5424R microcentrifuge. Subsequently, the supernatant was removed, the pelleted DNA origami resuspended in 1× FOB to 75% of the original volume, and incubated overnight at 30 °C at 600 rpm on an Eppendorf ThermoMixer C. The concentration of the purified DNA origami structure was estimated using Lambert–Beer’s law based on their absorbance at 260 nm using a BioTek Eon Microplate Spectrophotometer (Take3 plate, 2 μL sample volume, Supporting Information Note S10). The extinction coefficients for the different variants are listed in Table S4.

### Agarose Gel Electrophoresis

Agarose gel electrophoresis was used to evaluate the integrity of the NR after folding, purification, and the enzymatic activity assay, as well as to determine the success of fluorophore annealing. Furthermore, monitoring the shift in electrophoretic mobility allows to study the binding interaction between the DNA origami and the virus capsid proteins/AB-dendron conjugates. To this end, the samples were mixed with either 6× gel loading dye or 40% sucrose for samples containing a fluorophore and loaded onto a 2% (w/v) agarose gel (in 1 × TAE, 11 mM MgCl_2_). Ethidium bromide (EtBr) was used at a final concentration of 0.46 μg mL^–1^ to stain the DNA, and the gel was run for 45 min at 90 V in 1 × TAE buffer, supplemented with 11 mM MgCl_2_. The DNA was imaged under ultraviolet light, and if applicable, blue light (A488 channel) using a GelDoc XR+ or ChemiDoc MP system (both Bio-Rad).

### Transmission Electron Microscopy

Both plain and complexed DNA origami samples (2–4 nM) were prepared by deposition of a 3–5 μL droplet on a plasma cleaned (15 s oxygen plasma flash, NanoClean 1070, Fishione Instruments) Formvar carbon-coated copper grid (FCF400Cu, Electron Microscopy Sciences). Depending on the sample concentration, the droplet was incubated for 1.5–5 min, then the excess liquid was removed by blotting against filter paper. The samples were negative stained with 2% (w/v) uranyl formate solution (pH-adjusted with 25 mM NaOH) using a two-step procedure.^74^ The grid was first immersed in a 5 μL stain droplet which was blotted immediately, followed by immersion into a 20 μL stain droplet and incubation for 45 s. After a final blotting step, the grids were dried for at least 15 min. The imaging was performed either on a JEOL JEM-2800 electron microscope at an acceleration voltage of 100 kV or on a FEI Tecnai 12 Bio-Twin microscope at 120 kV.

### Nanoreactor Loading with Horseradish Peroxidase

HRP (Sigma-Aldrich) was covalently conjugated to DNA oligonucleotides (5′-thiol-modified, Integrated DNA Technologies) by using sulfosuccinimidyl 4­(*N*-maleimidomethyl)­cyclohexane-1-carboxylate (sulfo-SMCC), as described previously.^26^ Briefly, 250 μL HRP solution (1 mg mL^–1^ in 50 mM sodium phosphate buffer, pH 7.2) were mixed with 10 μL sulfo-SMCC (5 mg mL^–1^, No-Weigh sulfo-SMCC dissolved in deionized water, Thermo Fisher) and incubated on an Eppendorf ThermoMixer C for 2 h at room temperature (RT), 300 rpm. Simultaneously, 565 μL of the DNA oligonucleotide (*c* = 100 μM) were incubated with tris­(2-carboxyethyl)­phosphine (TCEP, 10× excess) for 2 h at RT, 300 rpm. The excess of sulfo-SMCC and TCEP, respectively, was removed by spin-filtration. To this end, a 10 kDa molecular weight cutoff (MWCO) centrifugal filter (Amicon) was washed with 50 mM sodium phosphate buffer pH 7.2 (5 min, centrifugation at 14,000*g*). 260 μL of the protein solution were added together with 260 μL of sodium phosphate buffer and centrifuged for 5 min, 14,000*g*, followed by three washing steps with 450 μL of buffer solution. The solution containing the DNA strand was first concentrated in a 3 kDa MWCO centrifugal filter (prewashed with 400 μL 1 mM EDTA solution) followed by two washing steps using 250 μL 1 mM EDTA solution (all steps 5 min, 14,000*g*). Both protein and DNA strand solution were collected by inverting the filter into a fresh tube using 2.5 min, 2000*g* and the respective buffer was added up to the starting volume. Finally, the HRP solution was mixed with the DNA oligonucleotide, resulting in a conjugation at ca. 6.5 μM using 10× excess of DNA oligonucleotides, and incubated for 30 min at 36 °C, after which the reaction tube was placed overnight at 4 °C.

To remove unreacted DNA oligonucleotides from the DNA-functionalized HRP, 200 μL of the reaction mixture were added into a 10 kDa MWCO filter (prewashed with 400 μL phosphate-buffered saline (PBS) buffer, 5 min, 14,000*g*) together with 200 μL PBS, 5 min, 14,000*g*. Subsequently, three washing steps with 400 μL PBS, followed by three washing steps with 400 μL deionized water were performed, 5 min, 14,000*g*. The purified protein was collected into a fresh tube by inverting the filter, 2.5 min, 14,000*g* and stored at −20 °C until further use.

For hybridization with the NR, the protein was used in 7.5× excess per available annealing site. The hybridization was performed in 1 × FOB at a final NR concentration of 7 nM by cooling the mixture from 40 to 20 °C with a gradient of −0.1 °C min^–1^. After storage for at least 4 h at 4 °C, the origami was diluted 1:1 with 1 × FOB, followed by 1:1 dilution with PEG precipitation buffer and a centrifugation step for 30 min at 14,000*g* to remove unhybridized HRP. The supernatant was removed, the pellet dissolved in 1× FOB to an estimated concentration of ca. 25 nM, and incubated overnight at 20 °C at 600 rpm.

### Buffer Exchange for DNA Origami

For complexation purposes, the DNA origami was transferred into 6.5 mM 4-(2-hydroxyethyl)-1-piperazineethanesulfonic acid (HEPES) buffer, supplemented with 2 mM NaOH (HEPES-NaOH, pH 6.5) using spin-filtration. To this end, a 100 kDa MWCO centrifugal filter (Amicon) was washed with 400 μL buffer (5 min, 14,000*g*). Then, the DNA origami solution and the HEPES-NaOH buffer were added in a 1:1 volume ratio into the filter, followed by a centrifugation step for 10 min at 6000*g*. The flow through was discarded, and HEPES-NaOH was added corresponding to 2.09× the initial volume of the DNA origami solution, and the centrifugation continued for 10 min. The flow through was used to dilute the sample, which was collected by inverting the filter into a fresh tube, and a centrifugation step of 2.5 min at 2000*g*, to ca. 25 nM.

### Complexation of Nanoreactor with Virus Capsid Proteins

The complexation with CCMV and MPyV CPs was performed similarly as reported by Seitz et al.^44^ (for virus preparation and capsid isolation (CCMV) as well as recombinant production of MPyV VP1 capsomers see Supporting Information Note S11). Briefly, for CCMV CPs the NR variants were first transferred into 6.5 mM HEPES-NaOH buffer using spin-filtration. Subsequently, the DNA origami and the protein solution (diluted in ‘CCMV clean buffer’, range depending on the molar ratio between CPs and DNA origami) were mixed in a 1:1 volume ratio, resulting in DNA origami concentrations of 4 nM (screening), 8 nM (enzymatic activity) or 6 nM (accessibility studies). The NaCl concentration was adjusted to 150 mM, resulting in a CCMV complexation buffer with 3.25 mM HEPES-NaOH, 25 mM Tris–HCl, 150 mM NaCl and 0.5 mM DTT. The samples were incubated at 4 °C for minimum 1 h, for enzymatic activity and accessibility studies, the complexation was performed ON.

For the complexation between MPyV VP1 and NR, the DNA origami was first diluted in 1 × TAE to decrease the MgCl_2_ concentration to 12.5 mM. Then, the DNA origami was added in a 1:1 volume ratio to the protein solution (diluted in ‘MPyV clean buffer’), resulting in a MPyV complexation buffer containing 40 mM Tris, 10 mM acetic acid, 1 mM EDTA, 100 mM NaCl, 6.25 mM MgCl_2_, 2.5% (v/v) glycerol and 2.5 mM DTT. The samples were incubated ON at 4 °C, and the used origami concentrations ranged from 4 nM (screening) to 6 nM (accessibility studies) or to 8 nM (enzymatic activity).

### Accessibility of Single-Stranded DNA on a Capsid Protein-Coated Nanoreactor

In order to study the accessibility of ssDNA on the surface of DNA origami complexed with CPs, 26 staple strands (edges, NR-E) or 18 staple strands (faces, NR-F) were exchanged to staples containing ss-overhangs (see Table S2) to facilitate annealing of a 5′-A488-labeled oligonucleotide (see Table S3). The coating of the NR variants was performed at a final DNA origami concentration of 6 nM (20 μL) and the samples were incubated ON at 4 °C. Subsequently, the A488-labeled oligonucleotide was added in 7.5× (NR-E) or 10.8× (NR-F) excess per annealing site and the samples were incubated ON at RT. To quantify the labeling yield, the CP coating was decomplexed by incubation with heparin, which acts as a competitive binding agent. To this end, 10 μL of heparin solution (*c* = 674 μM and 672 μM for NR-E and NR-F, respectively) were added for 15 min, followed by immediate loading of 10 μL of the solution onto a 2% agarose gel. To remove the excess of A488-labeled oligonucleotides 15 μL were mixed 1:1 with PEG precipitation buffer. After a centrifugation step at 14,000*g* for 30 min, the supernatant was removed, the pellet resuspended in 15 μL 1× FOB and the tubes placed on a shaker ON, RT, 600 rpm. The redissolved pellet was loaded on a 2% agarose gel. If not stated otherwise, unpurified samples were used for the evaluation of the accessibility by analyzing the fluorescence intensities of the bands using ImageJ and OriginPro2024b (OriginLab Corporation) and comparing them to an uncoated sample which served as a negative control. The significance was calculated from averaged relative triplicates with a two-tailed *t*-test.

### Enzymatic Activity Assays

The enzymatic activity of HRP was evaluated using the three different substrates oPD, TMB and ABTS at varying concentrations and in the presence of H_2_O_2_. The measurements were performed at a concentration of 2 nM DNA origami or free HRP using a sample volume of 80 μL. To this end, the samples were first complexed with the desired virus capsid proteins at 8 nM. Subsequently, the complexed samples were diluted 1:1 to 4 nM using complexation buffer; CCMV-coated samples were diluted with MPyV complexation buffer, MPyV-coated samples with CCMV complexation buffer to ensure comparable buffer conditions. The measurements were performed at pH 5, which was achieved by addition of sodium acetate at a final concentration of 5 mM. Moreover, after the samples were pipetted into a clear-bottom 96-well plate (Thermo Fisher Scientific), HCl was added to a final concentration of 0.0175%. Immediately before the measurement, 4 mM (final concentration) H_2_O_2_ was added, followed by the addition of the substrates. ABTS was dissolved into deionized water, while oPD was dissolved into 5 mM sodium acetate buffer and TMB into ethanol (Etax aa, 99.5 wt %, Altia Oyj), resulting in total 18.75% ethanol for measurements using TMB. Final concentrations of 0 mM, 0.125 mM, 0.25 mM, 0.5 mM, 0.75 mM, 1 mM, 2 mM or 4 mM (oPD), 0 mM, 0.25 mM, 0.5 mM, 0.75 mM, 1 mM, 1.5 mM, 2 mM or 4 mM (ABTS), or 0.075 mM, 0.15 mM, 0.225 mM, 0.3 mM, 0.5 mM, 0.75 mM or 1 mM (TMB) were added to the enzyme-containing solution, and the formation of the oxidized products was monitored by measuring the absorbance over time (15 s measurement intervals) using a BioTek Synergy H1 microplate reader. The oxidation product of oPD was read out at A_450_ for 30 min, whereas the activity for TMB as substrate was monitored at A_650_ for 30 min and for ABTS at A_420_ for 60 min. All the activity assays were performed as three independent replicates, i.e., independent enzyme annealing into the nanoreactor and purification, complexation and substrate preparation.

For the analysis of the enzyme kinetics, the Michaelis–Menten model was applied. First, the initial rate was determined by fitting the initial linear fraction of the recorded absorbance curves, representing the product formation over time. For oPD, 300 s starting from 3 min after the measurement began were used for fitting, for the comparison between NH and free HRP, 180 s were fitted, starting after 30 s. The kinetics for TMB were determined from 240 s (minutes 0.5–4.5, for comparison between NH and HRP the initial 105 s), and for ABTS from 300 s (4–10 min). Subsequently, *v*_max_ and *K*_M_ were determined by nonlinearly fitting the obtained initial rate vs substrate concentration plots. The fitting was performed in OriginPro2024b (OriginLab Corporation).

### Antibody-Mediated Plate-Based Fluorescence Immunoassay

The immobilization of the NH on a surface, which was mediated by the interaction between an anti-HER2 antibody fragment featuring a positively charged DNA binding site (aH, for preparation see Supporting Information Note S12) and the HER2 (ECD) receptor, was monitored based on the fluorescence intensity of the DNA origami. To this end, the edges (NR-E) were fluorescently labeled. Briefly, a 5′-A488-labeled oligonucleotide was added in excess (7.5× per annealing site; 26 sites) to the purified DNA origami (final *c* = 7 nM) and annealed by slowly (−0.1 °C min^–1^) cooling from 40 to 20 °C, after which the mixture was placed into the fridge for a minimum of 2 h. Subsequently, excess oligonucleotides were removed by diluting the fluorescently labeled NR (NA) 1:1 (volume ratio) first in 1× FOB, then in PEG precipitation buffer (15% (w/v) PEG8000 in 1 × TAE, 505 mM NaCl). After centrifugation at 14,000*g* for 30 min, the supernatant was discarded, the pellet was resuspended to a concentration of approximately 25 nM and the tube placed on a shaker ON at 20 °C.

The samples were prepared by first exchanging the buffer to 6.5 mM HEPES, followed by “complexation” with 0× excess CCMV at a final concentration of 4 nM. Subsequently, 15× excess of aH (in 10 mM HEPES, pH 7) was added, diluting NA to 3.2 nM. For the assay the complex is further diluted to 2 nM (220 μL), resulting in a final buffer composition of 40 mM Tris–HCl, 20 mM acetic acid, 1 mM EDTA, 1.25% (v/v) glycerol, 1.44 mM HEPES, 120 mM NaCl, 1.25 mM DTT, 3.25 mM MgCl_2_.

The HER2 receptor (ECD, Sino Biological) was diluted into 50 mM sodium carbonate buffer pH 9.6 to a concentration of 2 μg mL^–1^. 100 μL of the solution were pipetted per well (‘+HER2’) into a black 96-well plate (MaxiSorp, Thermo Fischer). As negative controls accounting for unspecific binding, wells without adding the receptor were prepared (‘–HER2’) After an ON incubation step at 4 °C to allow for the immobilization of the antigen, each well was first washed with 200 μL ‘washing buffer’ (1× PBS supplemented with 200 mM NaCl and 0.05% Tween 20) for four times, followed by a blocking step using BSA (150 μL per well; 1% BSA dissolved in 1× PBS supplemented with 0.05% Tween 20). The blocking solution was discarded after 2 h incubation at RT and the wells were washed four times with washing buffer and once with 1× PBS. 100 μL of the diluted sample was added to both the +HER2 and −HER2 well for 1 h at 37 °C, after which the wells were once again washed three times with washing buffer and once with 1× PBS. The readout was performed in 100 μL of 1× PBS pH 7.5 or pH 6 by exciting the samples at 480 nm, and recording the fluorescence spectra using a BioTek Synergy H1 microplate reader. The final fluorescence intensities correspond to the integrated spectra between 520 and 570 nm.

The activity of NH once immobilized was determined using oPD as substrate. To this end, the wells containing 100 μL 1× PBS pH 6 were mixed with phosphate-citrate buffer, pH 5.6, to a final concentration of 5 mM. Moreover, to enhance the fluorescence signal of DAP the buffer was supplemented with Triton-X^76^ (0.2%). oPD was added to final concentration of 0.125 mM, 0.25 mM, 0.5 mM, 1 mM, and 2 mM, and the enzymatic reaction was monitored for 30 min by measuring the fluorescence signal at 560 nm (excitation wavelength 428 nm).

### Susceptibility to DNase I Treatment

The stability of the (complexed) NR was tested in nuclease-rich environment. To this end, the DNA origami (final *c* = 3.2 nM) was incubated with DNase I (final *c* = 0–10 KU mL^–1^) for 15 min at 37 °C. To compensate for the high NaCl in the buffer (120 mM), the divalent ions MgCl_2_ and CaCl_2_ were added to the digestion reaction at final concentrations of 5 mM and 1 mM, respectively. AGE was used for detection. Note that the DNase I was not inactivated before gel loading.

## Supplementary Material



## Notes

Notes S1–S12 including characterization of the different NR variants using agarose gel electrophoresis and TEM, supplementary TEM images of CCMV and MPyV VLPs in assembled and disassembled state, supplementary TEM images of the NR encapsulated in virus CPs, supplementary agarose gels of unpurified samples assessing the surface accessibility of selected staple strands, disassembly of the CP-NR complexes using heparin, characterization of the loading of the NR (AuNPs, enzyme kinetics), supplementary kinetics data (product formation over time graphs, ν_max_ and *K*_M_ comparison of the different substrates), targeting of the NR (characterization of complexes of NR and p-*anti*-HER2 using TEM, immunoplate assay setup), DNase I stability assays, caDNAno design and staple list for the NR variants and supplementary methods including molar extinction coefficients for the NR variants as well as the preparation of the virus CPs and p-*anti*-HER2 (PDF)
