# Supplementary material for: Modular Virus Capsid Coatings for Biocatalytic DNA Origami Nanoreactors
Source: ACS Nano. 2025 Oct 8;19(41):36465–77. doi: 10.1021/acsnano.5c10734 (PMC12548342; doi:10.1021/acsnano.5c10734)
Supplement: Supplementary file 1 [file nn5c10734_si_001.pdf]

Supporting Information for
Modular Virus Capsid Coatings for Biocatalytic DNA Origami Nanoreactors

Iris Seitz[a], Donna McNeale[a,b], Frank Sainsbury[b], Veikko Linko[a,c] & Mauri A. Kostiainen[a,d*]

[a]Biohybrid Materials, Department of Bioproducts and Biosystems, Aalto University, Aalto 00076, Finland
[b]Centre for Cell Factories and Biopolymers, Institute for Biomedicine and Glycomics, Griffith University, Nathan, QLD 4111, Australia
[c]Institute of Technology, University of Tartu, Tartu 50411, Estonia
[d]LIBER Center of Excellence, Aalto University, Aalto 00076, Finland
[*]Correspondence and requests for materials should be addressed to mauri.kostiainen@aalto.fi

Contents

Page

Note S1: Folding of NR
(Supplementary Figure 1) S3
Note S2: Complexation of NR with virus capsid proteins
(Supplementary Figure 2–3) S4
Note S3: Surface accessibility
(Supplementary Figure 4) S6
Note S4: Disassembly of CP-NR complexes using heparin
(Supplementary Figure 5) S7
Note S5: Loading of NR with AuNPs
(Supplementary Figure 6) S8
Note S6: Enzyme kinetics
(Supplementary Figure 7–10) S10
Note S7: Targeting of NR
(Supplementary Figure 11–13) S15
Note S8: Treatment with DNase I
(Supplementary Figure 14) S16
Note S9: Design of the NR variants
(Supplementary Table 1–3) S16
Note S10: Estimation of DNA origami concentration
(Supplementary Table 4) S24
Note S11: Preparation of virus CPs
. . . . . S24
Note S12: Preparation of p-anti-HER2
. . . . . S25

Note S1: Folding of NR

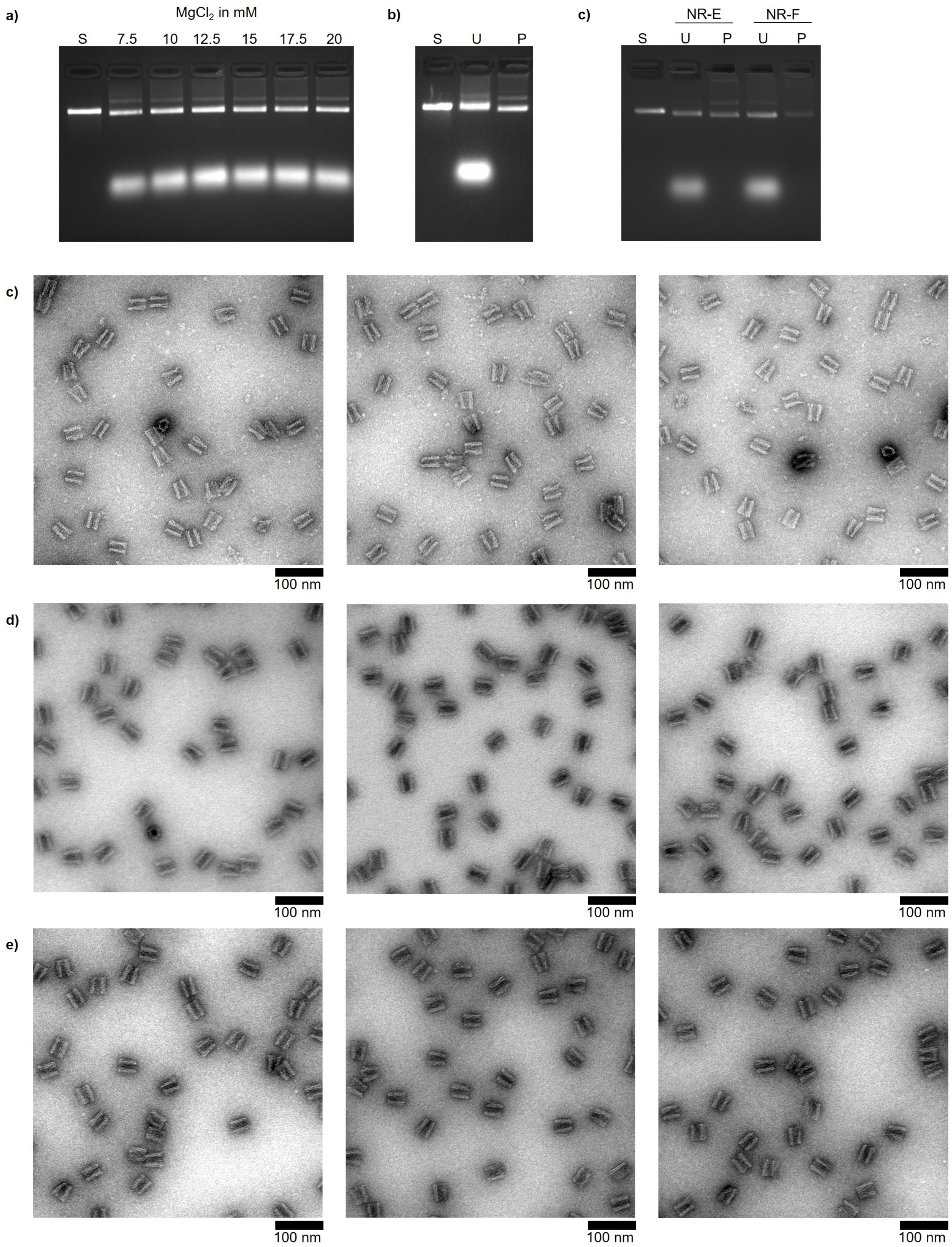

Figure S1. **a**, Optimization of the folding conditions for NR by screening the $MgCl_2$ concentration supplementing 1×TAE from 7.5–20 mM. **b**, A folding buffer of 1×TAE, supplemented with 15 mM $MgCl_2$ was chosen and the DNA origami structures successfully purified after folding. **c**, Folding and purification of NR-E and NR-F variants. The scaffold is denoted as S, unpurified samples as U and samples purified using PEG precipitation as P. **c–e**, Negative-stain TEM images showing the folding of NR (c), NR-E (d) and NR-F (e).

Note S2: Complexation of NR with virus capsid proteins

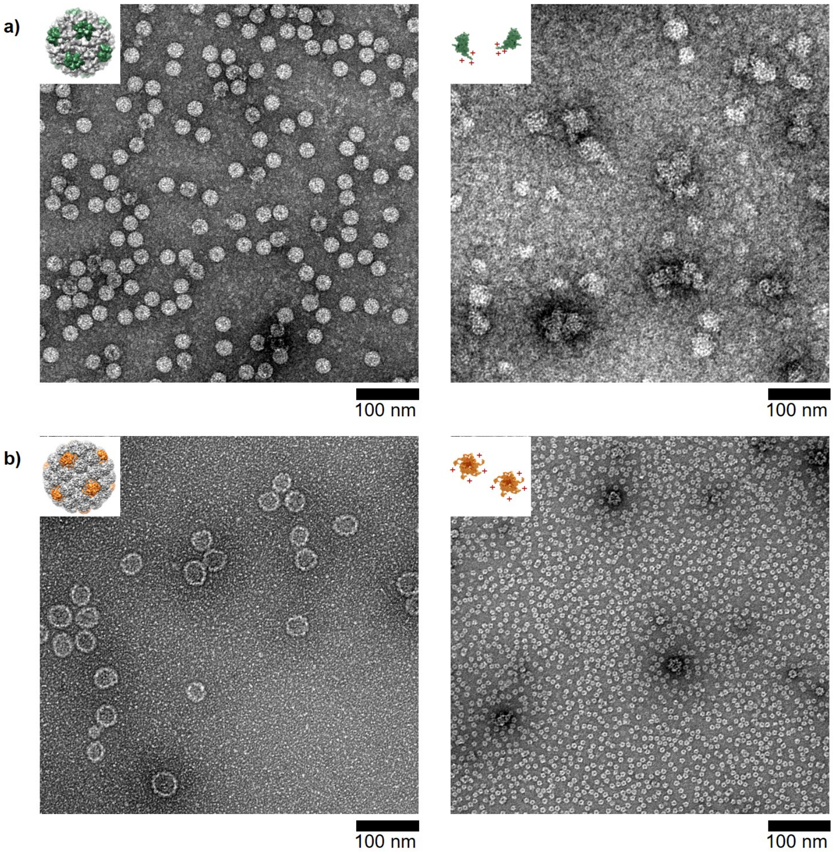

Figure S2. Supplementary negative-stain TEM images of the assembled (left) and disassembled (right) state of **a**, CCMV and **b**, MPyV VLPs.

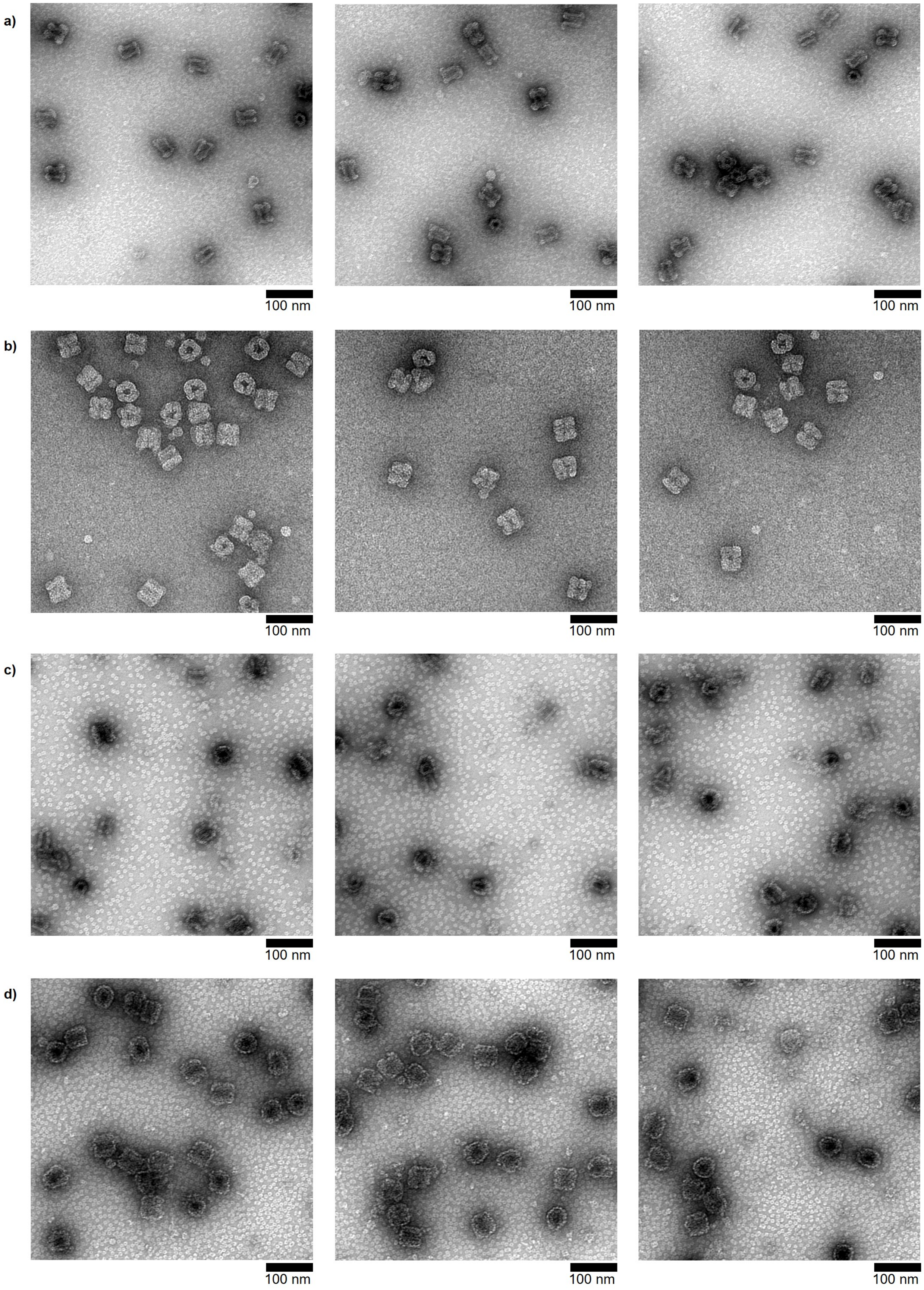

Figure S3. Supplementary negative-stain TEM images for **a**, NR-500C, **b**, NR-2kC, **c**, NR-500M, **d**, NR-1.25kM.

Note S3: Surface accessibility

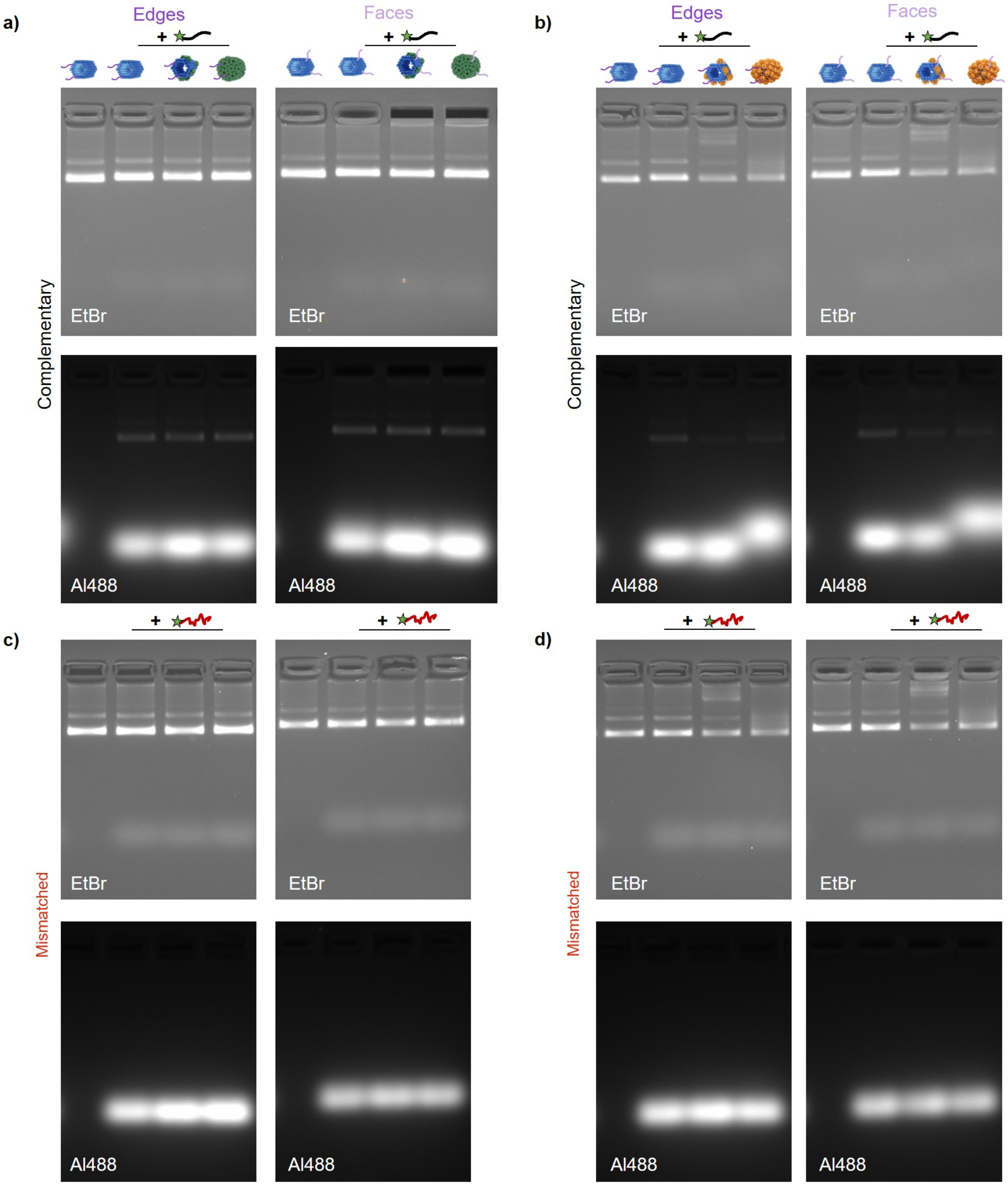

Figure S4. Supplementary, unpurified gels imaged under UV in the EtBr channel (top) and under blue light in the Alexa488 (Al488) channel (bottom). The fluorescence intensity was determined from unpurified gels, i.e., the excess of A488-labeled oligonucleotides had not been removed and could be detected as a strong band at the bottom of the gels. While the oligonucleotide bands were present in all gels, a leading DNA origami band could only be detected if ssDNA overhangs and A488-labeled oligonucleotide were complementary such as **a**, when complexed with CCMV CPs and **b**, when complexed with MPyV CPs, which was not the case for **c**, complexes with CCMV CPs and **d**, complexes with MPyV CPs.

Note S4: Disassembly of CP-NR complexes using heparin

Heparin served as a competing agent to disassemble the complexed NRs by facilitating the disintegration of CPs. The amount of heparin used is NR variant dependent and expressed as the ratio between $n_{sulphates}$ and $n_{phosphates}$. Using the assumption of 2.33 sulfate groups per repeating IdoA(2S)-GlcNS(6S) disaccharide unit and an average molecular weight of 17–19 kDa results in an average of 71 negatively charged sulfate groups per heparin molecule (1). The negative charge of each NR, originating from the phosphate groups in the DNA backbone, was estimated to 15952 and 15904 for NR-E and NR-F, respectively, under the assumption that all possible annealing sites were hybridized to A488-labelled oligonucleotides. An electrophoretic mobility shift assay (EMSA) was used to monitor the release of the NR variants from the CPs (Figure S5). To ensure full release, 250× ratio is used for both structures for CCMV as well as MPyV CPs.

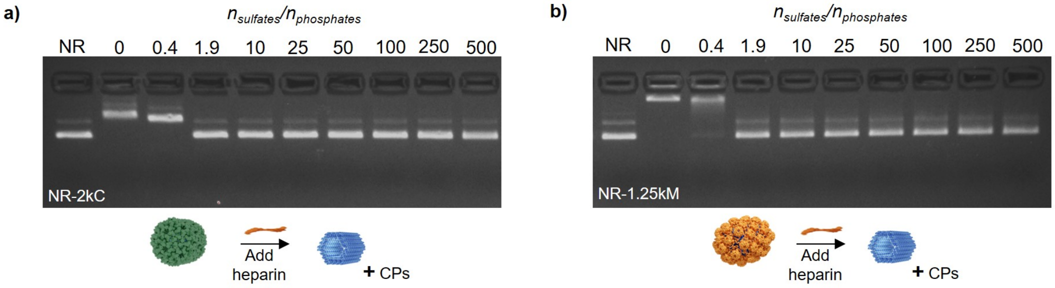

Figure S5. Heparin was used to release the DNA origami from **a**, encapsulation with CCMV CPs and **b**, a protein shell made from MPyV CPs.

Note S5: Loading of NR with AuNPs

Gold nanoparticles (AuNPs) were used in order to confirm the accessibility of the inner cavity of the reactor. To this end, four staple strands (see Table S2) were exchanged to overhang-containing strands in the folding reaction. These should later facilitate the hybridization to DNA-functionalized AuNPs (or DNA-functionalized HRP) which were added to the purified NRs in 30× excess (7.5× per available annealing site) for a reannealing step (c_{NR} = 7 nM) by heating to 40 °C and subsequent cooling to 20 °C using a gradient of -0.1 °C min^{-1}, followed by incubation at 4 °C for at least 2 h.

The oligonucleotide-functionalized AuNPs were prepared under constant shaking (600 rpm, Eppendorf ThermoMixer C) at 40 °C, similarly to previously described procedures (2, 3). Briefly, 80 µL of citrate stabilized AuNPs (5 nm, 100 nM, Sigma Aldrich) were incubated with 1.6 µL 1 % sodium dodecyl sulfate (SDS) for 20 min, followed by the addition of 8 µL of the thiol-modified oligonucleotide (100 µL, Integrated DNA Technologies). After 30 min, salt-aging was performed by sequentially adding 2.5 M NaCl in intervals of 5 min. First, 0.8 µL were added 6×, then the amount was increased for 6× to 1.6 µL, followed by 5× 3.2 µL and a final addition of 4 µL. Then, 120 µL of 1×FOB supplemented with 0.02 % SDS were added to the AuNPs and incubated for 60 min. The incubation was continued ON, however, the temperature was decreased to 20 °C.

Unbound thiol-modified oligonucleotides were removed from the functionalized AuNPs before use by spin-filtration. First, 100 kDa MWCO filters were washed with 400 µL 1×FOB, supplemented with 0.02 % SDS, in a centrifugation step for 5 min at 14,000*g*. Subsequently, 460 µL of oligonucleotide-functionalized AuNPs were added into the filter and upconcentrated for 10 min at 14,000*g*, followed by the addition of 230 µL of oligonucleotide-functionalized AuNPs together with 240 µL of 1×FOB, supplemented with 0.02 % SDS. Centrifugation was continued and the particles washed 3× with 200 µL 1×FOB, supplemented with 0.02 % SDS. The oligonucleotide-functionalized AuNPs were collected by inverting the filter into a fresh tube (5 min, 1,000*g*).

After the annealing, non-hybridized AuNPs were removed by PEG precipitation. 12.5 µL of PEG buffer (17.5 % (w/v) PEG 8000 in 1×TAE, supplemented with 500 mM NaCl and 10 mM $MgCl_2$ were mixed with 50 µL of DNA origami solution and incubated for 10 min at 4 °C. After centrifugation for 30 min at 12,600*g* and 4 °C, the supernatant was removed, the pellet resuspended in 1×FOB, supplemented with 0.02 % SDS, and incubated ON at 20 °C, 600 rpm. To confirm the accessibility of the ssDNA overhangs, the sample was visualized under negative-stain TEM.

At first, the accessibility of each of the ssDNA overhangs was examined separately, *i.e.*, NR-variants with only one ssDNA overhang (A1–D1) were used (Figure S6). AuNPs were present in the cavities of all NR-variants, confirming the accessibility of the ssDNA overhangs, however, the loading yield was low. By exposing all four annealing sites simultaneously, the yield of NR carrying one AuNP increased significantly (Figure S7). Therefore, the NR-variant with four ssDNA overhangs was used for further experiments (NH), unless otherwise stated.

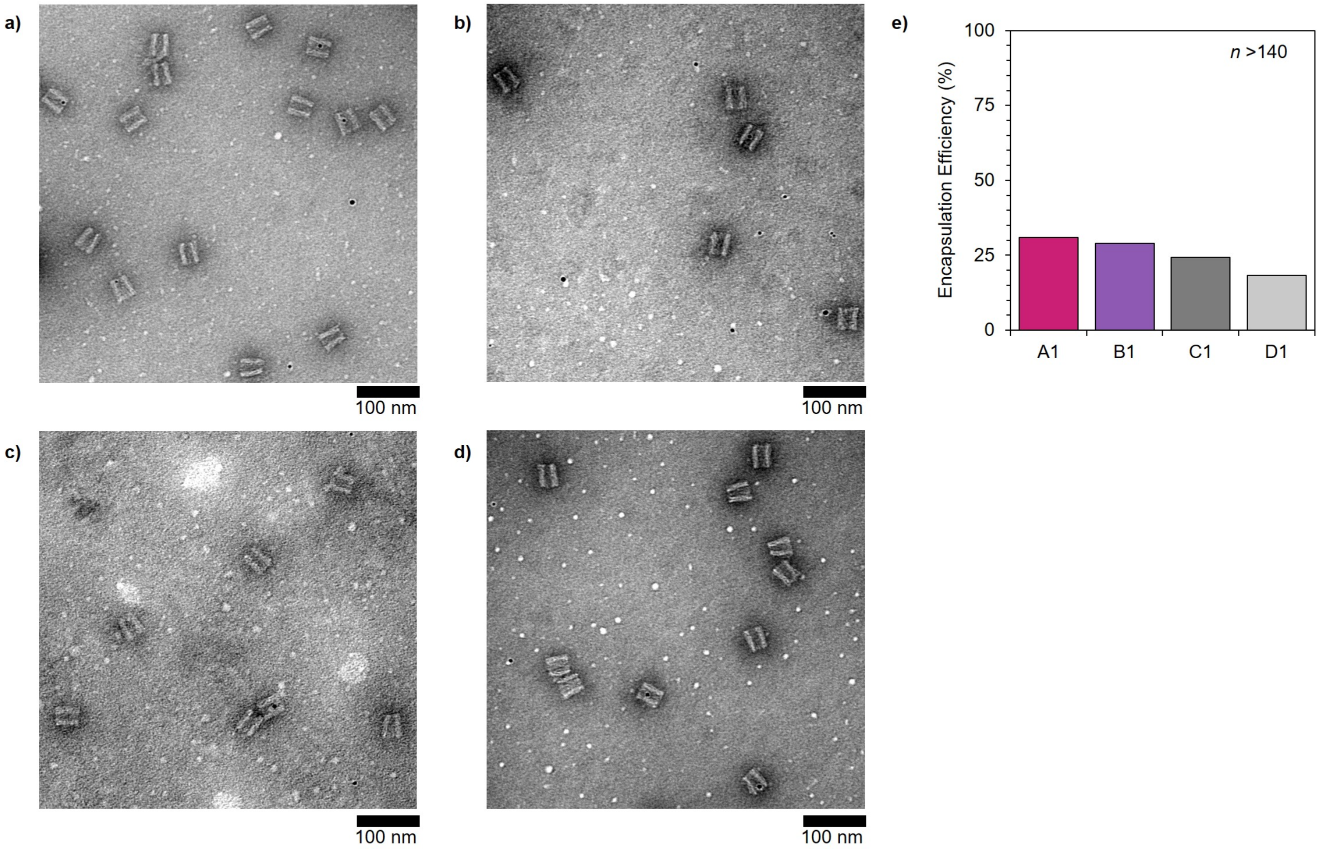

Figure S6. Negative-stain TEM images showing AuNP-loaded NR-variants with a single ssDNA overhang protruding in the cavity labeled **a**, A1, **b**, B1, **c**, C1, and **d**, D1. **e**, Statistical analysis of the loading yield of the NR-variants.

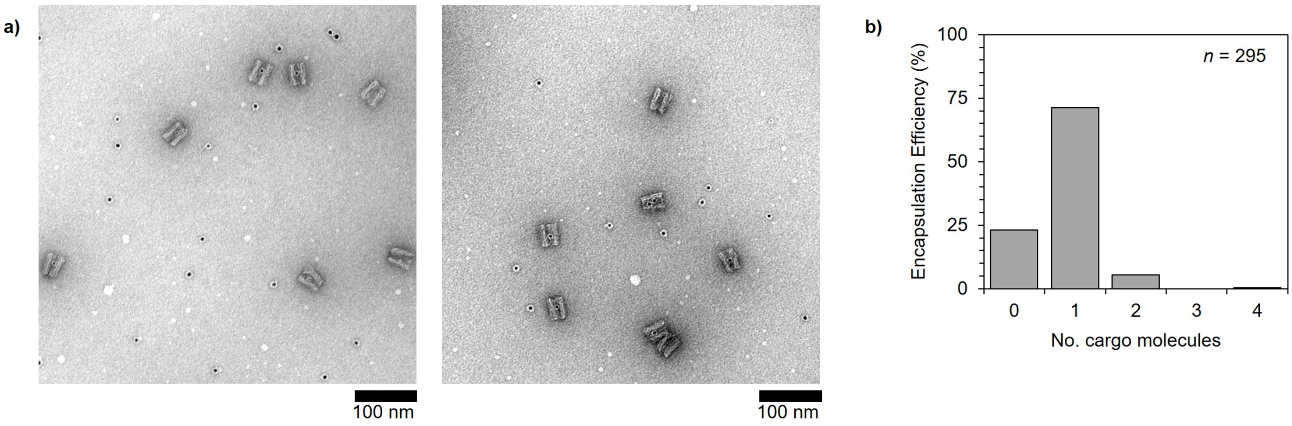

Figure S7. **a**, Negative-stain TEM images of AuNPs loaded into the cavity of NH (four ssDNA overhangs protruding in the cavity) and **b**, statistical analysis of the number (no.) of cargo molecules located inside the NH, confirming the accessibility of the cavity and showing a loading yield for AuNPs of ca. 75 %.

Note S6: Enzyme kinetics

To study the enzyme kinetics, three different substrates were used, oPD, TMB and ABTS. In the presence of H_2O_2, oPD is oxidized to 2,3-diaminophenazine (DAP) by HRP, resulting in a color change of the solution from colorless to orange-brown (absorbance at 450 nm). Similarly, TMB is oxidized to 3,3',5,5'-tetramethylbenzidine diimine (TMBox), and ABTS to an $ABTS^{\bullet+}$ radical. As a result, the solution turns either blue (TMBox) or green ($ABTS^{\bullet+}$), which allows the product formation to be followed at A_{650} nm and A_{420} nm, respectively.

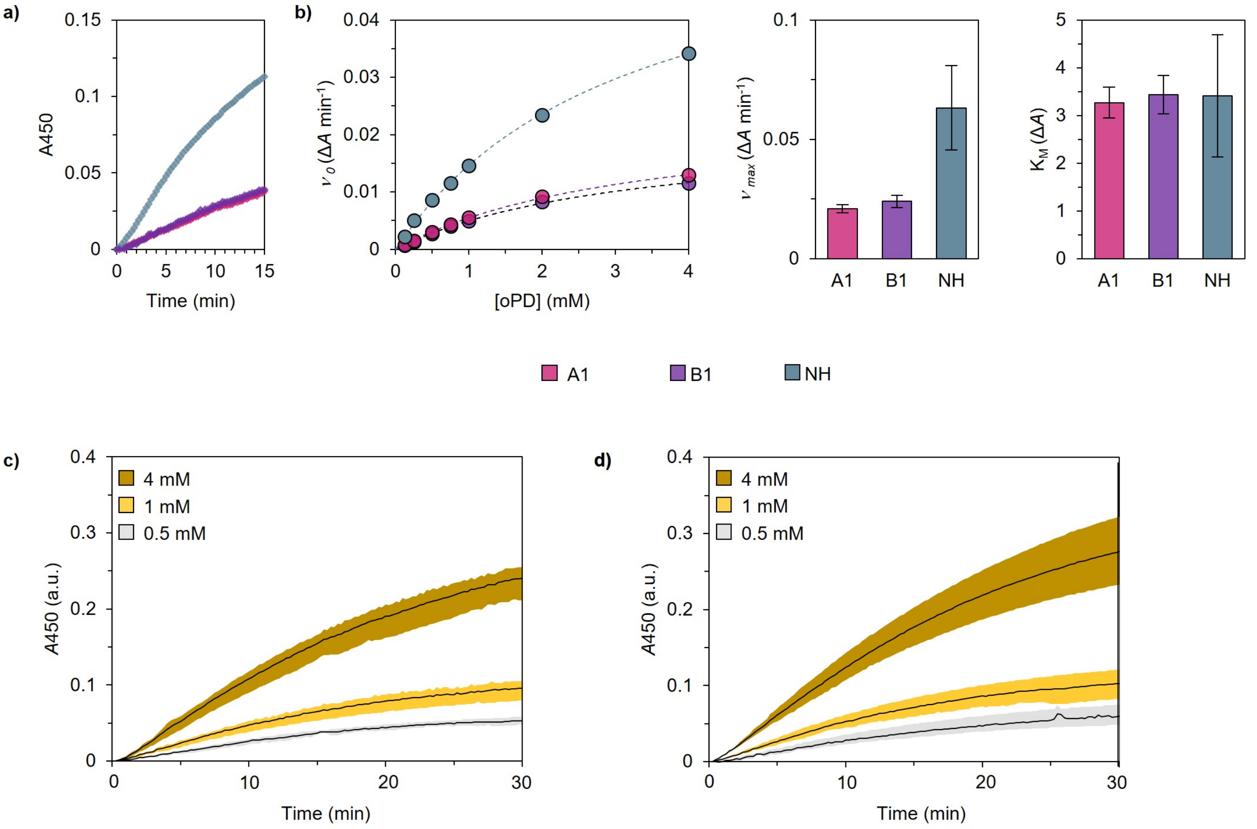

Figure S8. Impact of enzyme load on the activity of the catalytic NR. A1 (pink) and B1 (purple) have one ssDNA oligonucleotide to load the enzyme whereas NH (blue) has four, yielding a higher enzyme load. **a**, Absorbance over time monitoring the oxidation of oPD (0.5 mM) in the presence of H_2O_2. **b**, Initial rate vs. substrate concentration-plot (left), from which $\nu_{\max}$ (middle) and K_{M} (right) were determined. **c–d**, Supplementary graph showing the product formation over time for A1 (c) and B1 (d) for oPD starting concentrations of 0.5 mM (grey), 1 mM (yellow) and 4 mM (dark yellow). The absorbance is presented as the average of triplicate measurements, and the range between minimum and maximum values is colored.

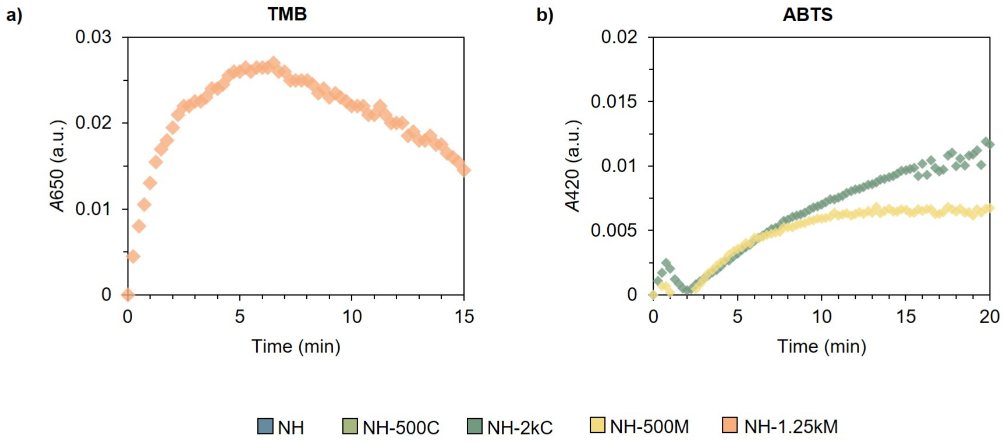

Figure S9. Supplementary absorbance spectra for **a**, TMB and **b**, ABTS showing the low absorbance region and the product formation in NH-1.25kM samples (orange) as well as in NH-2kC (dark green) and NH-500M (yellow) samples.

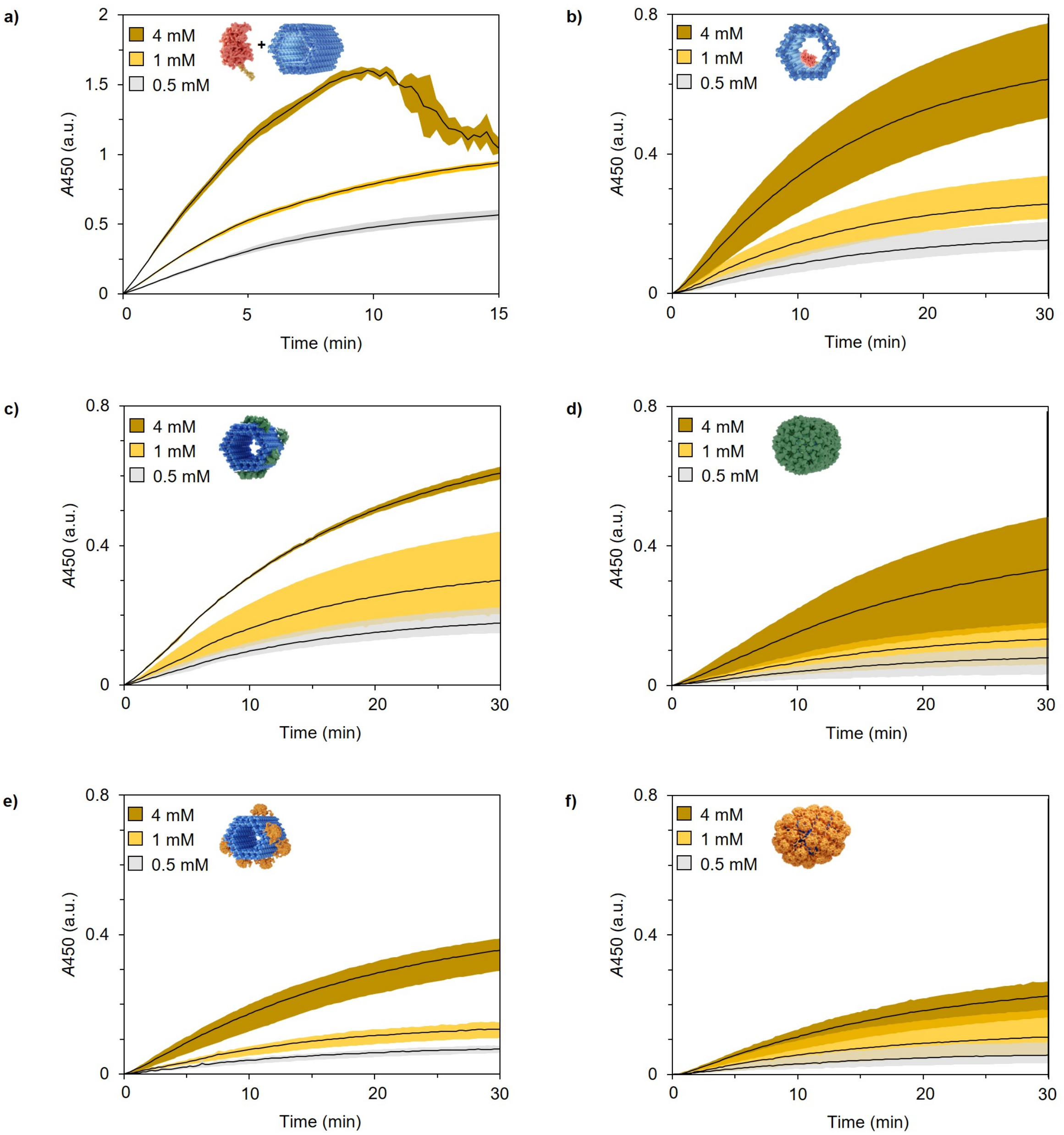

Figure S10. Supplementary graphs showing the product formation of DAP over time in the presence of H_2O_2 and **a**, DNA-functionalized HRP, **b**, NH, **c**, NH-500C, **d**, NH-2kC, **e**, NH-500M and **f**, NH-1.25kM. oPD starting concentrations of 0.5 mM (grey), 1 mM (yellow) and 4 mM (dark yellow) are shown. The absorbance is presented as the minimum and maximum values of triplicates measured, together with the calculated average (black line).

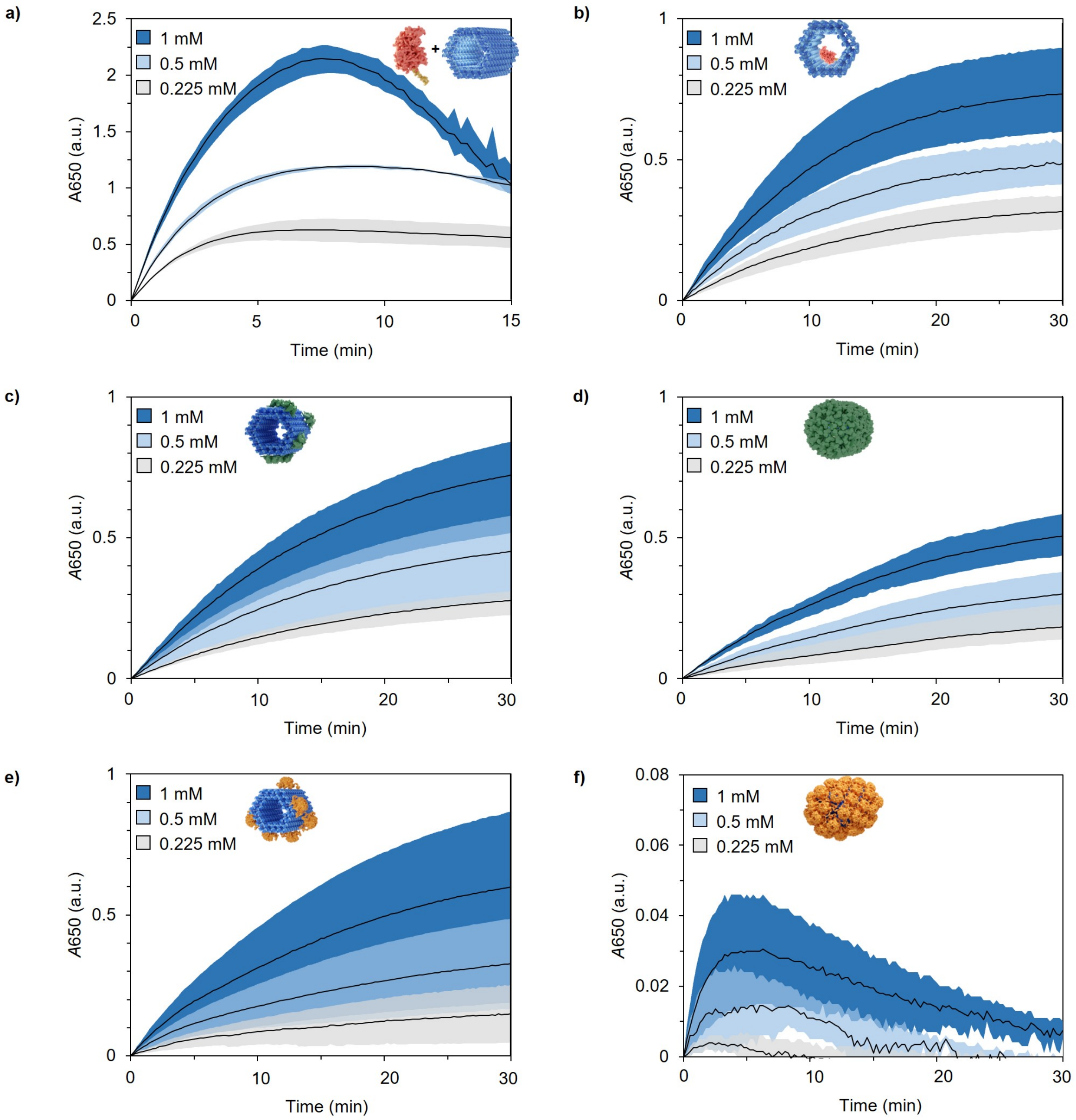

Figure S11. Supplementary graphs showing the product formation of TMBox over time in the presence of H_2O_2 and **a**, DNA-functionalized HRP, **b**, NH, **c**, NH-500C, **d**, NH-2kC, **e**, NH-500M and **f**, NH-1.25kM. TMB starting concentrations of 0.225 mM (grey), 0.5 mM (light blue) and 1 mM (dark blue) are shown. The absorbance is presented as the minimum and maximum values of triplicates measured, together with the calculated average (black line).

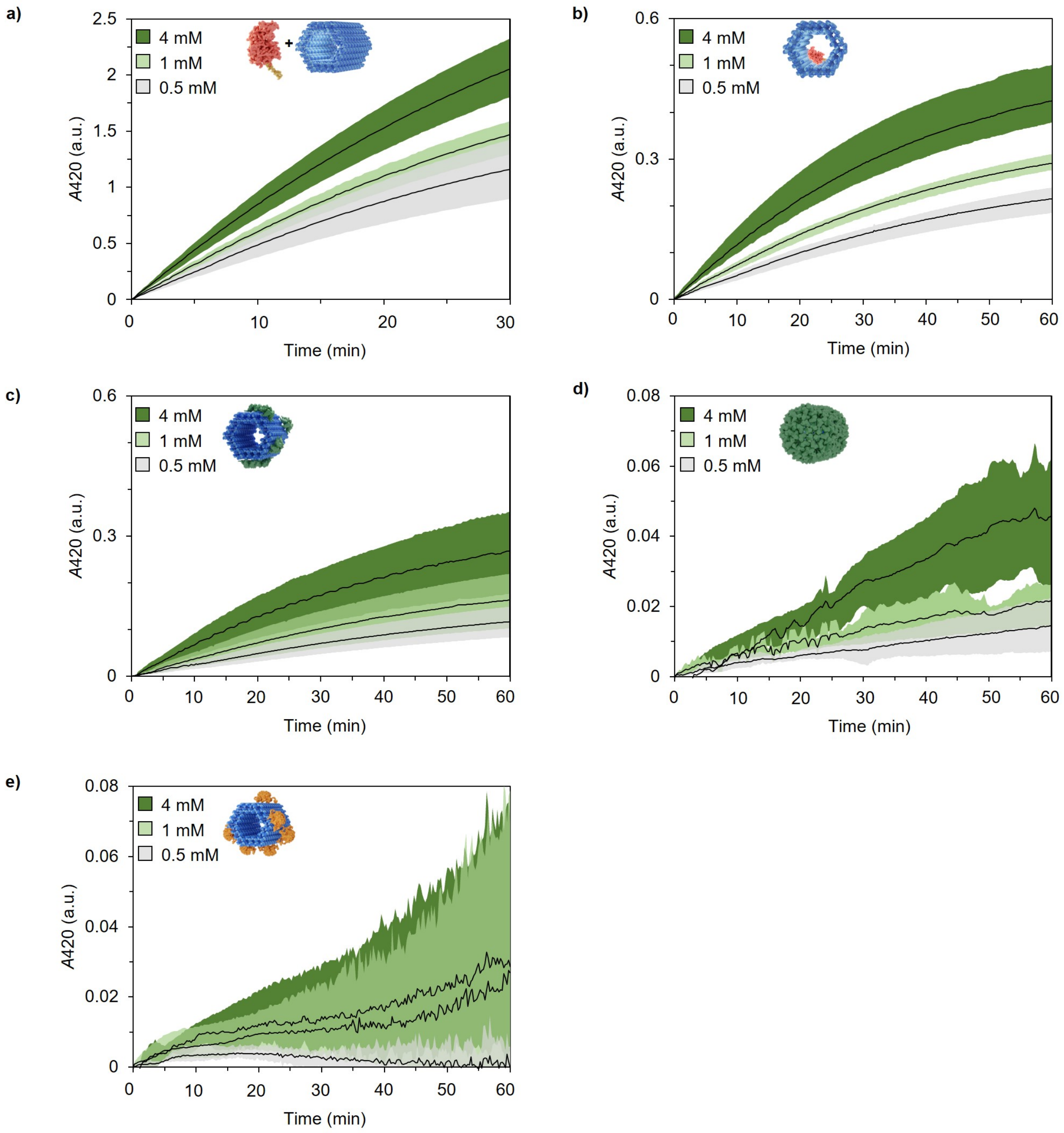

Figure S12. Supplementary graphs showing the product formation of $ABTS^{\bullet +}$ over time in the presence of H_2O_2 and **a**, DNA-functionalized HRP, **b**, NH, **c**, NH-500C, **d**, NH-2kC, **e**, NH-500M. The catalytic activity of NH-1.25kM was not determined. ABTS starting concentrations of 0.5 mM (grey), 1 mM (light green) and 4 mM (dark green) are shown. The absorbance is presented as the minimum and maximum values of triplicates measured, together with the calculated average (black line).

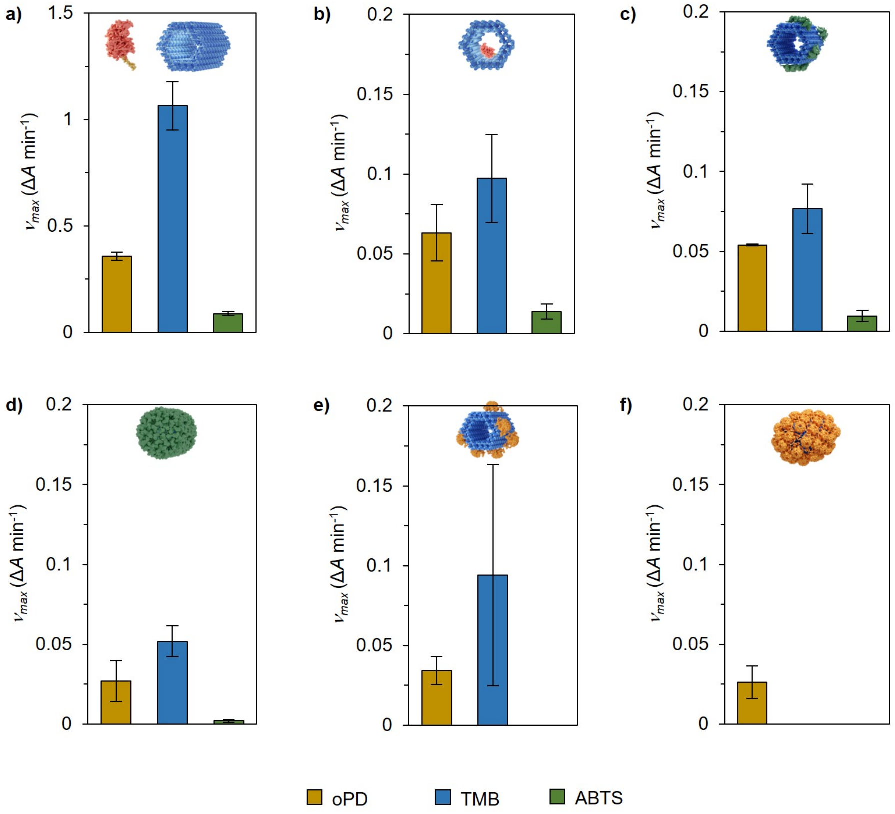

Figure S13. Comparison of ν_{max} determined for oPD (dark yellow), TMB (blue) and ABTS (green) as substrates for **a**, DNA-functionalized HRP, **b**, NH, **c**, NH-500C, **d**, NH-2kC, **e**, NH-500M, and **f**, NH-1.25kM.

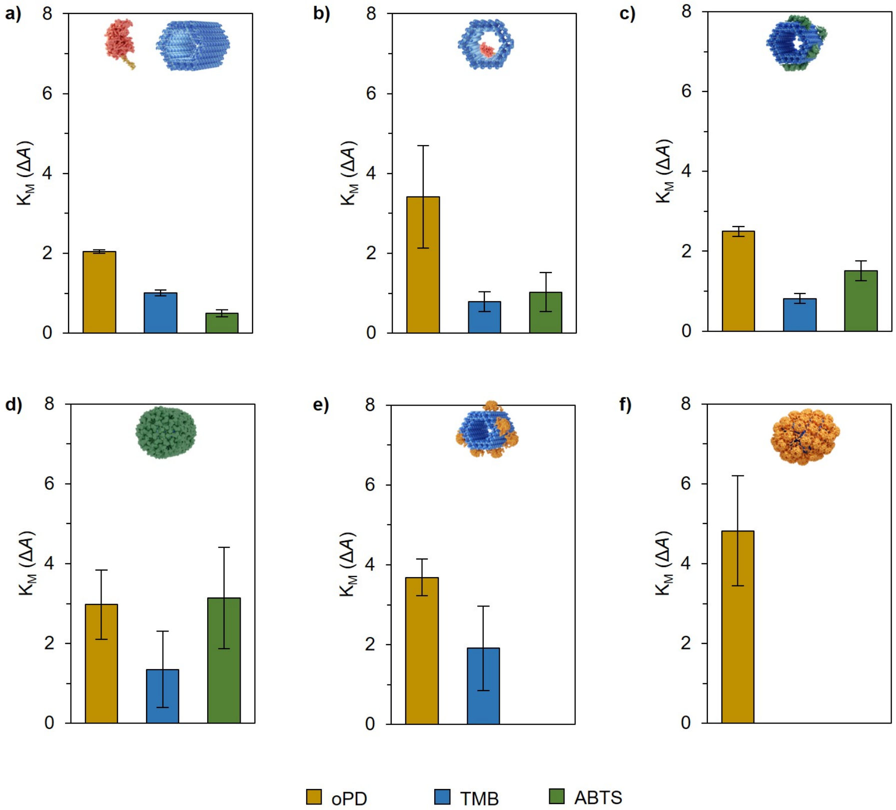

Figure S14. Comparison of K_{M} determined for oPD (dark yellow), TMB (blue) and ABTS (green) as substrates for **a**, DNA-functionalized HRP, **b**, NH, **c**, NH-500C, **d**, NH-2kC, **e**, NH-500M, and **f**, NH-1.25kM.

Note S7: Targeting of NR

p-anti-HER2 (aH, Figure S15), consists of the targeting moiety anti-HER2 and the artificial binding domain, which is a second generation Newcome-type dendron (G2) with a positive charge of +27 due to the spermine surface groups (4). The core *N*-maleimido group allows for simple coupling to the sulfhydryl group of a free cysteine residue in the protein (inset), which is located at its *C*-terminus, resulting in the formation of a covalent bond (see Note S12).

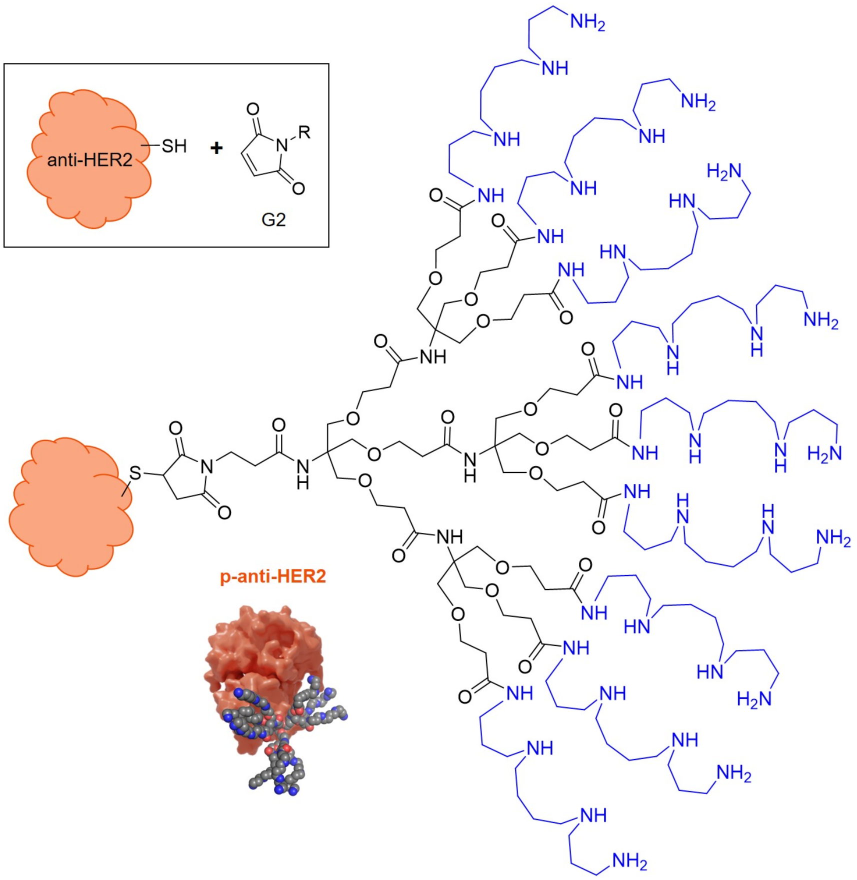

Figure S15. Schematic representation and the chemical structure of p-anti-HER2. It is prepared by forming a covalent bond between a free cystein residue and a *N*-maleimido group (inset).

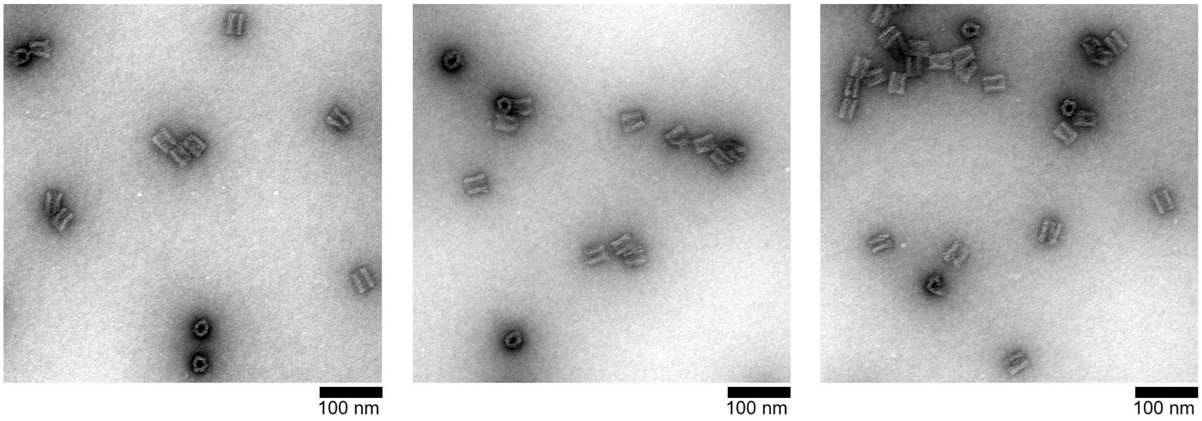

Figure S16. Supplementary negative-stain TEM images of NR-E complexed with 15× excess of aH.

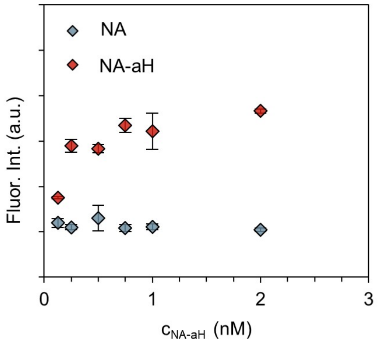

Figure S17. Different DNA origami concentrations (NR-E variant labeled with A488-labeled oligonucleotides, NA) were tested to determine an optimal concentration for the binding assay (constant HER2 concentration 2 µg mL^{-1}). Already at 0.25 nM a clear difference between samples complexed without (blue) or with 15× excess of p-anti-HER2 (NA-aH, red) could be observed. To resemble concentrations used for HRP assays, 2 nM was chosen as the final DNA origami concentration for the immunoassay.

Note S8: Treatment with DNase I

Treatment of NR-E (Figure S18a) with increasing concentrations of DNase I shows a clear digestion at 2.5 KU mL^{-1}. In comparison, despite a small shift in electrophoretic mobility, a leading band could be detected for NR-E complexed with CCMV CPs at c_{CP}/c_{NR} = 150 at 10 KU mL^{-1}, whereas no visual signs of degradation could be detected for fully encapsulated structures (c_{CP}/c_{NR} = 2.5k) Note, that the complexes have not been disassembled, nor was DNase I inactivated.

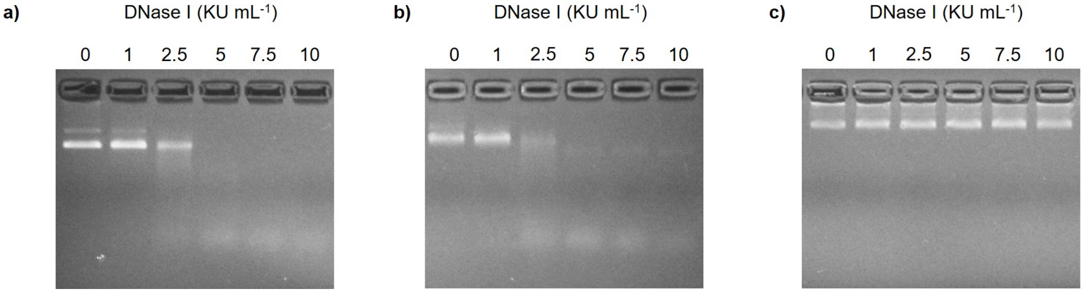

Figure S18. AGE was used to monitor the degradation of the (complexed) NR when increasing the concentration of DNase I. **a**, NR-E, **b**, NR-E complexed with CCMV CPs at c_{CP}/c_{NR} = 150 and **c**, fully encapsulated NR-E (c_{CP}/c_{NR} = 2.5k).

Note S9: Design of the NR variants

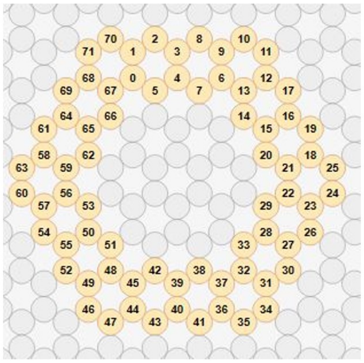

Figure S19. Cross-section of the NR

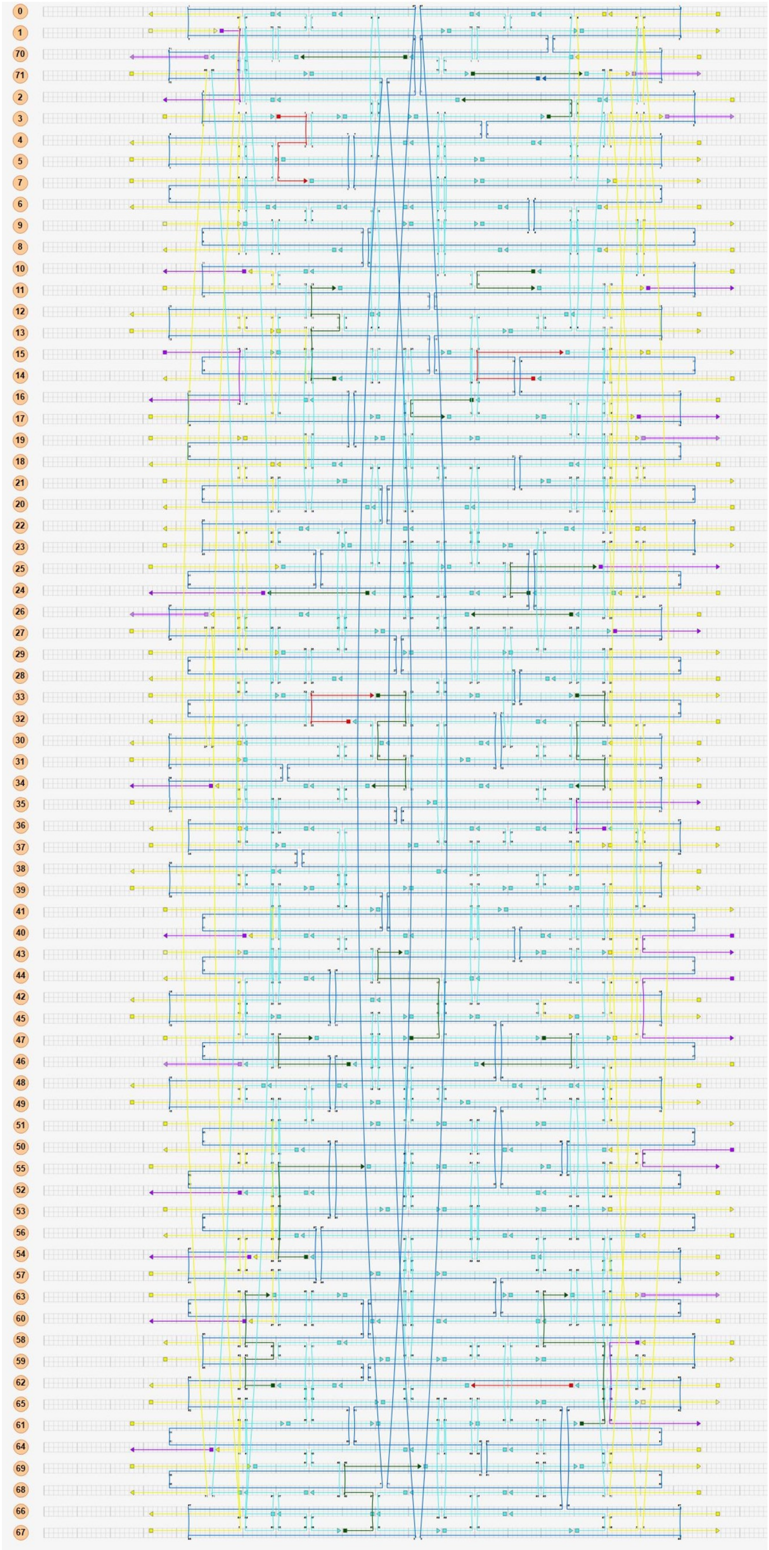

Figure S20. Blueprint of the caDNAno design of the NR variants. The core staple strands are marked in light blue, strands for poly-T passivation in yellow and purple. The purple sites can be exchanged to facilitate annealing of A488. Red and green staples can be exchange to allow the annealing of DNA-functionalized HRP and A488 (NR-F), respectively.

Table S1. Staple sequences for folding the NR.

Functionality	Start	End	Sequence
core_1	50[118]	50[98]	CAGTTGGCACGACTTAAGTGT
core_2	53[72]	50[54]	CCCGCCTTATGACAATGTCCCGAACGAA
core_3	24[120]	24[103]	ACATAACGCCAAAAGGAA
core_4	67[84]	68[77]	GAAGGGACACTATTAAAGAAC
core_5	30[41]	26[35]	TATATTTCTCAAATCGTCATA
core_6	63[98]	64[98]	AAGAACTCCATCACGAGAGAGATGGTGG
core_7	29[50]	26[55]	ATTAAGTACCCTGACAGTTCAGAAAACTAGCGTCC
core_8	57[84]	56[98]	GTAAACAGGGCTCATGGAAATGGGGCCT
core_9	17[85]	12[70]	TCCCGACCTGAACAAATAACCGATATA
core_10	54[103]	55[105]	ACACGACCAGTAAGGTTGTGAATTCATG
core_11	35[82]	34[93]	AGAAGAGTCAATAGTGAATTTATTATATAACTATA
core_12	61[70]	64[77]	AGCTGATTGCCCTTCTCATTA
core_13	39[77]	45[90]	ATAACGGATTCGCCAAACCACTCAGCTC
core_14	27[71]	25[84]	CCATAATCGCCATTAGTAAAAATAGCGCAACACTGGAACAAC
core_15	59[105]	53[118]	AACGGTACGCCAGATGGGCACATGATAC
core_16	65[105]	62[112]	ACATGCCGCCGCGCTGCTTTG
core_17	22[76]	29[76]	GAAAAAACGAGTAGCCGAAAGACTTCAA
core_18	15[85]	20[69]	AAAGAAGATTTTTTGGAGGCTTTGAGGAAAACGAAAGAACCG
core_19	19[71]	24[69]	CAAAGTTGTCTTTAACTAACATCATAAC
core_20	40[105]	41[96]	ACGGTAACAGTAACAAGGGTA
core_21	46[91]	51[83]	CATTCAGGCTGCGCATCGCTATAACGCCACTGAGAG
core_22	20[68]	14[62]	GATATTCATTACCGAAGGCACCAACCTACTAAAGA
core_23	69[113]	61[112]	AAATCAGAAAATCCTGTTTGTTGCAGCA
core_24	17[105]	18[112]	TGAAGCCTTAAATCTTTTATCGTCGAAACGAGGCG
core_25	1[113]	2[118]	GCCGTAAAGCACTACCAATGA
core_26	68[97]	67[111]	AAGAGTCAGAAAGCGAAAGGAGCGGGCG
core_27	10[55]	17[69]	TTTCGAGTGCGCCGCTTGCAGCCGGTATTCTAAGAACGCGAG
core_28	49[89]	48[100]	CAGCTGGTAGATTAGAAATTC
core_29	43[105]	44[91]	AAAACTAGAAGATTGTATAAGCAAATAT
core_30	2[87]	3[77]	CGACAGAATGCCCGGAATAGCCACCA
core_31	25[85]	21[105]	ATTATTAATCAATCCTAATTTACGAGCATCATAAG
core_32	33[91]	33[111]	TAAAGCCAAATAAGAGGTCAT
core_33	37[50]	37[77]	GGGACAATTTCATTTGAATTACCTTTTT
core_34	34[55]	36[42]	ACGCGTTCTACTAAAGGCAAGGGTTGTA
core_35	22[54]	23[63]	GCTTGATTACCTTATAGACGA
core_36	65[71]	62[62]	CAGTCTAGAGCGGGAGCTAAC
core_37	5[92]	0[104]	GGGAGGGAAGGTAAATATTGGAGCTTGA
core_38	8[61]	6[48]	TAATTTTTTCACGAGCCTTTTAAGACTC
core_39	66[97]	61[90]	ACAGGAGTGTACTGTACCGTTATAAATCCACCGCC
core_40	6[76]	13[97]	ACCCAAAGCAACGGCTACATTTAACGTCAAAAATG
core_41	44[90]	47[104]	TTAAATTTTTGAGGGGACGACGACAGTA
core_42	30[92]	30[72]	TTTAGTTTGACCATTAGATAC
core_43	19[42]	18[49]	AAGCAAGCCGTTTTGAACGGG
core_44	0[103]	1[112]	CGGGGAAGACTGTAGCGAGGT
core_45	15[49]	21[62]	CCACTACCAAATCACTTGCCCTGACGAG
core_46	36[104]	39[111]	CCTTGAATCAATATATGTGAGCCTGAGCACCAAGT
core_47	0[61]	1[84]	CTCAAGTCGAGAGGGTTGATATAAGTATACAAGTT
core_48	30[71]	32[65]	ATTTCGCAACAAATATCGTGT
core_49	40[55]	36[56]	AAAGGGTATATGATATTCAACCATAAAG
core_50	23[98]	26[112]	AAGAAAAGGTAAAGTAATTCTTATAAAG
core_51	64[97]	65[104]	TTCCGAACCAGTAAGCGTCAT
core_52	18[111]	24[91]	CAGACGGTCAATGTAGAAACCACAGGTAAGGCATA
core_53	34[92]	31[88]	TGTAAATGCTGATGCATTAATTTCATCT
core_54	2[48]	71[55]	AGGTTTAGCGGATATAATCAACCTCCCTCAGAGCC
core_55	61[91]	59[104]	TGGCCCTGCAAATTGGATTTTAGACAGG

Functionality	Start	End	Sequence
core_56	45[112]	43[118]	TTGCCCGAAAACAGGCATGTC
core_57	29[98]	23[97]	TTCAAAGTTATCAACAATAGATTTGCAA
core_58	51[105]	48[112]	AAAGCATCACCTTGTAGACTT
core_59	19[91]	16[91]	TATCATCGCCTGATAAATTGTCTGAATCTTACCACACTCATC
core_60	69[80]	68[98]	GATATTCACAAACAAATCGGCAAAATTGGAAC
core_61	44[55]	40[56]	GGGTTAGACAGAAATAAAGAAAGATTCA
core_62	4[103]	5[91]	GCCATTTGGGAATAGCAAGCCCGATTGA
core_63	26[89]	33[90]	GGGTAAATTTAACGTAGGGCACCAGTA
core_64	60[69]	63[62]	ATAAAGACGGAGGATGAAATT
core_65	61[51]	59[71]	CACCACCACACATGGGGTGCCTAATGAGTGAGCTA
core_66	41[97]	40[106]	GCTATTTTTGAGAGACGATGA
core_67	42[69]	39[55]	TTATCATCATATTCCTGATTATTAAATG
core_68	17[70]	16[56]	GCGTTTTATTATACCAAGCCGTAGGAAT
core_69	52[104]	55[83]	AACAACTAACGAAAGGGGGATGTGCTGCATGTTCT
core_70	12[69]	6[63]	TTCGGTCGAGGGTAAGAACTG
core_71	48[62]	51[69]	CGTCTGGACCGCCTGCAACAG
core_72	49[70]	47[76]	GGCCTCTACTGTTGCGTGCAT
core_73	18[61]	19[70]	TTCCAATATTTTCATGCGAAA
core_74	20[89]	15[84]	GAGTAATCTTGACAAGAGGCA
core_75	16[55]	15[48]	CATTACCGCGCCCACGTAATG
core_76	29[77]	29[97]	ATATCGCGTTTTAATTCGAGC
core_77	9[42]	3[48]	CAAAAGGTTGAAAACCGCCAC
core_78	8[117]	10[104]	TCAACAGACGTTAGTAAATGAGAGCAAGAAACAATGAAATAG
core_79	45[49]	48[63]	ACTTCTGAATAAATTCGCATTTAATTCG
core_80	25[50]	18[62]	AAATCTACGTTAATAAAACGCCTTATCA
core_81	12[110]	6[97]	CGGGAGCAGCCTTAGTTTTGTCCCGAGG
core_82	36[55]	34[56]	CTAAATCGCAAAGAATTAGCAACAAAGA
core_83	60[104]	60[84]	AAAAACGCTTAAGCTACGTGG
core_84	45[77]	42[70]	TGTTAAACAGAAGGAGCGGAA
core_85	57[70]	54[56]	GGTTGGTTGGCCAACAGAGATAGAACCC
core_86	34[68]	35[81]	CGCAAGAAATTAAGCAATGACGCTG
core_87	66[69]	67[62]	CGGGGTCAGTGCCTTATTATT
core_88	56[97]	53[104]	TGAATCGCGCTCGCCCTGGAG
core_89	48[111]	52[105]	TACAAACGCCGTCAGAGCACT
core_90	38[83]	39[76]	CATCAAGAAAACAAAATTAATTGAGTAATGAAACA
core_91	21[63]	19[90]	AAACACCAGATAATATCCCATAATCGGCACAACGGAGATTTG
core_92	0[90]	4[77]	CGAACGTGGCGATTAAGAGGCAGGGCGACATTCAACCAATAG
core_93	43[42]	46[42]	ACGTAAAAACCTACACGTTGGCGGATTG
core_94	7[92]	4[104]	AAACTACAACGCCTGTAGCACGACTTGA
core_95	52[55]	49[69]	GCCAGGGCGGATTCTCCGTATCGGTGCG
core_96	3[78]	8[62]	CCCTCACAACTAAAGGAATTGCGAATAA
core_97	55[106]	47[119]	CGCAAATCAACAGTCTTTAGATAGATACCGGAAACAGGAAGA
core_98	47[57]	46[65]	GGCGCATCGTAACGGAAG
core_99	9[92]	8[97]	CCAGAAGGAAAGTCTTTCCAGTTTCAGCGGAGTGA
core_100	66[48]	62[49]	CAGTGCCAACCTGTCGTGCCATAATTGC
core_101	56[53]	53[71]	TCTTTATTAAAAATACCGCCAAAATAAC
core_102	6[47]	5[49]	CTTATTACATAAACACGGAACATATGGT
core_103	15[110]	22[111]	AGCGTCTTTAGGCGCAGAACTGACAGAACGC
core_104	50[53]	48[47]	CCACCTATTAACCCTTCCTGT
core_105	6[96]	7[91]	AAACGCAATAATAACCAGTAC
core_106	39[112]	36[119]	TACAAAAAGAGAATTCTACAAAGGCTATTAATTAA
core_107	60[83]	63[97]	TGCTTGTTAACATCACTTGCCTGAGTAG
core_108	13[98]	17[104]	AAAATAGAATTAACTGAACACCTTGCGGGAGGTTT
core_109	58[62]	61[69]	CACAATTGTGAGACGGGCAAC
core_110	21[106]	20[90]	GGAACCTAGGCTGGCTGACCTTCATCAA
core_111	50[97]	51[104]	CCTTAGTCAAATGAAAAATCT

Functionality	Start	End	Sequence
core_112	68[76]	67[83]	GTGGACCATGAAAGTAGAAAG
core_113	11[104]	12[111]	AATATCAGAGAGATAGCATTAGA
core_114	7[56]	6[77]	ACATATAAACGTAACACTGAGTTTCGTCACGGAAT
core_115	69[44]	61[50]	TGACAGGAGGTTCCAACGCGCGGGGATTTTCTTTT
core_116	2[117]	7[119]	AACCATCCAGTAGCCGTCACTTCCACAG
core_117	4[76]	3[62]	GAACCCATGTACAGAAACGCAACCGCCA
core_118	22[125]	19[125]	GCTAATGCCAACTTGCCGGAATCCGCGA
core_119	62[61]	65[70]	TCACATGCTGCATTAAAAGCG
core_120	38[48]	45[48]	CTTTATTATATATTTCAGATGGGATTAT
core_121	43[76]	43[104]	TCAGGTTTAACGTCAGATGAATATATCGT
core_122	8[96]	3[105]	GAATAGAAAGGAATTTTCAGGGATTAGAGCCAGCA
core_123	34[111]	36[105]	GTTGGGTCAAAATCTTAGAAT
core_124	31[89]	28[107]	TCTGACCATGCAACTACGAACGAACATGTAATTTAGGTTGCT
core_125	28[61]	22[55]	GGTCTTAGGAAGCTAAATTGG
core_126	32[104]	38[84]	GTTTTAAATTAAATTTAATGGAAACAGTAAACAAA
core_127	11[62]	9[91]	ACAACAACCATCGCCCACGCAGTCAGAGGACCGAAGCAAAGTTA
core_128	59[84]	58[63]	ATTAAAGAACCGTTGTAGCAATACTTCTTTCCGCT
core_129	63[48]	57[69]	TTCCTGTGTCCCCGGGAATGGCTACTCTGACCTCCT
core_130	5[50]	0[62]	TTACCAGCGCCAAAGACAAATGAGACTC
core_131	59[72]	56[54]	AACAGCATAAATCATTTCTCCGAATTAG
core_132	41[70]	40[77]	GCTGATAAATTAATGCCGGAGGTACCTTTTACATC
core_133	63[111]	54[104]	ATCGGCCTGCCATTGTTTTGACGCAGATTCACCAGTC
core_134	6[117]	14[104]	ATCTAATACAGAGATTATTTATCCCAAT
core_135	67[112]	66[98]	CTAGGGCCCACCACACGCTTTTGATGAT
core_136	1[85]	71[89]	TGCCTTTTTTCGGAGGGCG
core_137	68[41]	70[35]	CCACCAGGGAACCGAATCACC
core_138	53[84]	59[83]	CTATTTAGCTGACGCATTTCAGAGGCCG
core_139	48[46]	52[42]	AGCCAAACCCGTTTTTCCC
core_140	33[50]	37[49]	CCTGTTGGCGTTAAATAAGAACTTTTGC
core_141	71[56]	70[77]	GCCACCCTCAGAACCGCTCCAACGTCAATCATAGC
core_142	62[89]	66[70]	CCTCGTTAGAATCCTGAATTGTAATAAGTTTTAA
core_143	23[64]	27[70]	CGATAAAAACCAAATGTTTAGACTGGAGAGAATGA
core_144	55[84]	57[83]	TCTAAGTTAAAAGGGACATTCGTAATGA
core_145	51[84]	53[83]	CCAGCAGGCTGAATTGTCAACTTCTAAT
core_146	48[99]	49[88]	GACATTAACCAATAGGTACGC
core_147	26[54]	33[49]	AATACTGCGGAATGCTTTAAACTATTAAGAAAAAG
core_148	30[118]	32[105]	GATTCCCAATTCTGAAGTACGTCAACAT
core_149	0[48]	68[42]	TTAGGATCCTATTTAGAGCCG
core_150	64[76]	68[56]	AAGCCAGAATGGATGAATCGGGAGGCAGGAGCCAC
core_151	56[125]	65[125]	TCGTCGGATCCTGACTATGGTTTAATGC
core_152	31[42]	30[42]	TGGCATCAAAGAAAACTTTTTATGGTCAATAACCTGTTTAGC
core_153	36[90]	41[69]	CGATAGCTTAGATTAAAAAGCCTCAGAGCGTTCTA
core_154	6[62]	9[76]	GCATGATAATTGTATCGGTTAAGCAGAT
core_155	9[77]	10[56]	AGCCGAACCCTTTTTAAGAAAAGTTATCAGCTTGC
core_156	40[76]	44[56]	GGGAGTGTAGGTAAATTGCGTTTTGTTAAATGGAA
core_157	70[111]	0[91]	GGGGTCGCGTTTTCATCGGCATAGCGTCAAGCCGG
core_158	22[110]	27[119]	GCCTGTCGAACCAGACCGGACCTTTAACAGAGGCA
core_159	3[63]	2[49]	CCCTCAGAGGTGTATCACCGTACTCAGG
core_160	24[90]	22[77]	GTAAGAGAGAGGCTTAAGTCCTGAACAA
core_161	70[53]	0[49]	TTTCAAGTGCCGAGAAGGA
core_162	63[63]	60[70]	GTTATGATTAGTAATACCTCG
core_163	37[78]	28[62]	TAATGGTTTGAAAAATTCTTTTAATTGAGAAATCAAAAATCA
core_164	45[91]	39[97]	ATTTTACTCGTATTTTTGCGGAACAAAGTGATTGC
core_165	39[56]	38[49]	CAATGCCTACATTTAAGAAGC
core_166	71[114]	69[112]	CCCACAGTGTTGTTCCAGTTCCCTTAT
core_167	55[68]	52[56]	CCAGGGTGGAAGGCGATTAAGTTGGGTAAC

Functionality	Start	End	Sequence
core_168	53[105]	60[105]	TGACTCTGAATATAACCTACACAACAGG
core_169	51[70]	45[76]	TGCCACGTCAAAAAAAATTTT
core_170	28[106]	30[93]	CCTTTTGCGCTCAACAAACGCCAGTAGA
core_171	39[98]	36[91]	TTTGAATAAAAGAAGATGATGACATAAAAACATAG
core_172	68[55]	66[49]	CACCCTCCGGAACCTGAGTAA
core+A_1	46[64]	47[56]	GGCGGGGAACAAACGGTGTAGATG
core+A_2	10[103]	11[103]	CAATAGCTATCTTGTAATTGAGCGCT
core+A_3	33[112]	34[112]	TTTTGCGCTGTAGCGTGTCTGGGCTTAG
core+A_4	26[111]	26[90]	TACCGACAAGTTTTGCCAGAGG
core+A_5	14[61]	11[61]	CTTTTTTCGGAACGCTGAGGACAATG
core+A_6	67[63]	69[79]	CTGAAACACCCTCAGTCAGACGATTGGCCTT
core+A_7	70[76]	70[54]	CCCCTTATTAGCGTTTGCCATCT
core+A_8	33[70]	34[69]	TTATACATACCGACATTTTAGAATCCAAT
core+A_9	61[113]	63[110]	AGCGGTGTAAAAGAGTCTGTCAAACT
core+A_10	71[90]	71[113]	AAAAACCGTCTATCAGGGCGATGG
core+A_11	62[48]	63[47]	GTTGCGCTAAAGCCACATACGAGCTGT
core+A_12	3[106]	2[88]	AAATCACGATAGCAGCACCGTAATCAGTAG
core+A_13	47[77]	43[75]	CTGCCAGGTAAACGTTAATATAGATTT
core+A_14	24[102]	25[116]	TTACGGAAAGATTCATCAGTTGAG
core+A_15	54[55]	55[67]	TTCTGACCAAGCTTTCTCAGGAGAAG
core+A_16	47[105]	46[92]	TCGGCCTCCAGGCAAAGCGCCATTCGC
core+A_17	16[90]	17[84]	TTTGACCCCCAGCGAGCGAACC
core+A_18	24[68]	24[47]	CCTCGTTTACCGCGATTTTAAG
core+H_1	14[103]	15[109]	CCAAATAAGAAACTACACTAAAAACGCTAACG
core+H_2	32[64]	33[69]	GATAAATAATAGTATCATATGCG
core+H_3	3[49]	7[55]	CCTCAGAAAGACACGGTGGCA
core+H_4	62[111]	62[90]	ACGAGCACGTATAACGTGCTTT
polyT_1	31[18]	31[41]	ttttttttGCGCGAGCTGAAAAGG
polyT_2	12[138]	11[126]	ttttttttAAACAGGGAAGCACCCACAA
polyT_3	60[142]	63[125]	ttttttttAACAATATTACCGCCATGCTGGT
polyT_4	17[22]	13[48]	ttttttttTCAGATATAGAAGGCTTATGGAGTTAAGCGAAA
polyT_5	39[18]	38[18]	ttttttttAATTTTTAGAACCCTCTCAACGCAAGGATAAAtttttttt
polyT_6	34[138]	31[138]	ttttttttTTTTTAACCTCCGAAGTTTCATTCtttttttt
polyT_7	47[120]	45[138]	TCGCACAGCCCCAAACGTTATTAATtttttttt
polyT_8	4[138]	8[118]	ttttttttGGTGAATTATCACACCATTAACAACTT
polyT_9	7[120]	7[138]	ACAGCCCTCATtttttttt
polyT_10	52[142]	50[119]	ttttttttAAGGTTATCTAAAATATTGAAAGATCTGGT
polyT_11	71[18]	68[18]	ttttttttGCCACCACCAACCACCACtttttttt
polyT_12	61[18]	64[36]	ttttttttTGGGCGCCAGGGTGGTGAGGCG
polyT_13	65[22]	66[22]	ttttttttTTCCAGTCGGGACGTATAAACAGTtttttttt
polyT_14	5[18]	4[18]	ttttttttCAATCAATAGAAAATTTAAGTTTATTTTGTCAtttttttt
polyT_15	23[25]	29[49]	ttttttttCTTTAATCATTGTGAAGATGGTTTCAAAAAG
polyT_16	46[145]	49[138]	ttttttttTCCGGCACCGCTTCTGGTGATACATTTGAGGtttttttt
polyT_17	56[145]	56[126]	ttttttttTCTGTAAGCAAC
polyT_18	19[22]	19[41]	ttttttttCTCATCGAGAAC
polyT_19	47[25]	44[25]	ttttttttGGATAGGTCCATATCAAAtttttttt
polyT_20	51[25]	54[44]	ttttttttGAGGTGAGGCGGTCAGAGCAGAACCAGTGCCTGAA
polyT_21	14[145]	13[138]	ttttttttTTACAAAATAAACAGCCATAGAATAACATAAtttttttt
polyT_22	42[138]	45[111]	ttttttttTTTAAAAGTTTGAGTAACATTATCATAAATCCT
polyT_23	3[25]	8[25]	ttttttttCCCTCAGAATCTCCAAAAtttttttt
polyT_24	41[25]	40[43]	ttttttttAGTCAAATCACCATCAGAGAAA
polyT_25	13[49]	14[25]	GACAGCACATGAGGAAGTTTCCATTAAACGtttttttt
polyT_26	69[18]	69[43]	ttttttttCAGAGCCGCCGCCAGCAT
polyT_27	9[25]	9[41]	ttttttttAAAAGGCTC
polyT_28	45[18]	42[18]	ttttttttATAATCCTGATTGTTTATGGCAATTCATCAATtttttttt
polyT_29	33[22]	28[22]	ttttttttATCATAATTACTTAGTCAGAAGCAtttttttt

Functionality	Start	End	Sequence
polyT_30	28[142]	29[142]	ttttttttTCAGGATTAGAGAGTAAGCAAACTCCAACAGGtttttttt
polyT_31	25[22]	25[49]	ttttttttAGTCAGGACGTTGGGAAGAA
polyT_32	22[145]	22[126]	ttttttttACAACATGTTCA
polyT_33	16[142]	17[124]	ttttttttTTGCACCCAGCTACAAAAGATT
polyT_34	43[25]	43[41]	ttttttttATTATTTGC
polyT_35	68[138]	71[123]	ttttttttGAGATAGGGTTGTACGT
polyT_36	35[18]	34[36]	ttttttttATCCAATAAATCATACTAGTAG
polyT_37	38[138]	37[118]	ttttttttGAATTATTCATTTCAATTATGAATAA
polyT_38	13[18]	12[18]	ttttttttGATCGTCACCCTCAGCAAGGCCGCTTTTGCGGtttttttt
polyT_39	0[142]	67[142]	ttttttttCCCTAAAGGAGTGTAGCGtttttttt
polyT_40	6[138]	6[118]	ttttttttAGTTAGCGTAACG
polyT_41	18[142]	21[145]	ttttttttTGTTACTTATGAAAGAGGACAtttttttt
polyT_42	21[25]	18[22]	ttttttttCATTCAGTGCCAAGTACCGCAtttttttt
polyT_43	37[22]	32[22]	ttttttttTGACCCTGTAATATAAACACCGGAtttttttt
polyT_44	54[142]	57[142]	ttttttttTGGATTATTTACATTGGCTCAATCGTCTGAAAtttttttt
polyT_45	26[138]	24[121]	ttttttttGTAATAAGAGAAGTCCAGACAGAT
polyT_46	62[142]	59[145]	ttttttttGGGCGCGTAGAAGTGTTTTTAtttttttt
polyT_47	0[118]	5[138]	CGATTTAACGGAAATTATTCATTAAAtttttttt
polyT_48	7[18]	6[18]	ttttttttAACGTAGAAAATACATACGCAGTATGTTAGCAtttttttt
polyT_49	27[18]	30[18]	ttttttttTGAATCCCCTCATTTGGGtttttttt
polyT_50	43[119]	39[138]	AATCATAGCAAACATCGCGCAGAGGCtttttttt
polyT_51	53[25]	60[43]	ttttttttGCCCTAAAACATCGCCAATGCGCTATTTTTGTACCG
polyT_52	1[22]	1[36]	ttttttttGCTCAGT
polyT_53	29[22]	22[25]	ttttttttAAGCGGATTGCATAATTTCAAtttttttt
polyT_54	37[119]	33[142]	CCTTGCTTATAATGGATGGCTTAGAGCTTAtttttttt
polyT_55	65[126]	65[142]	GCCGCTACAtttttttt
polyT_56	55[22]	50[25]	ttttttttGTAAAACGACGGGATAAAACAtttttttt
polyT_57	63[22]	58[25]	ttttttttAATCATGGTCATAGCCGGAAGtttttttt
polyT_58	30[138]	30[119]	ttttttttCATATAACAGTT
polyT_59	10[145]	9[145]	ttttttttAGCCCAATAATAAATTTTCTGTATtttttttt
polyT_60	20[145]	15[126]	ttttttttGATGAACGGTGTACAGACCCCAGAGCC
polyT_61	48[138]	51[145]	ttttttttATTTAGAAGTATCTGAACCTCAAATATCAAAtttttttt
polyT_62	32[142]	37[142]	ttttttttATTGCTGAATCTGTAAATtttttttt
polyT_63	66[142]	0[119]	ttttttttGTCACGCTGCGCGTAAGCTGGCAGAGCCCC
polyT_64	58[145]	58[126]	ttttttttTAATCAGTGAGG
polyT_65	36[41]	36[22]	CCAAAAACATTAtttttttt
polyT_66	8[145]	3[130]	ttttttttGGGATTTTGCTAACCAT
polyT_67	36[142]	41[145]	ttttttttCGTCGCTATCAGGTCATTGCCtttttttt
polyT_68	59[25]	62[22]	ttttttttCATAAAGTGTCACTGCCCGCTtttttttt
polyT_69	64[138]	69[138]	ttttttttGCCCCAGCAGGCAAAGAATAGCCCtttttttt
polyT_70	24[142]	23[145]	ttttttttCAACTAATGCGACGACAATAAtttttttt
polyT_71	57[22]	56[25]	ttttttttTGGCACAGACAAGAACTGATAtttttttt
polyT_72	18[48]	20[25]	TATTAAAAATAAGGACGTAACAAAGCTGCTtttttttt
polyT_73	2[145]	1[142]	ttttttttGCCGGAAACGTCAAATCGGAAtttttttt
polyT_74	67[22]	0[22]	ttttttttTAATGCCCCCTGTAGCGGGGTTTTtttttttt
polyT_75	53[119]	53[145]	CGACAGTGCGGCCCTGCCAtttttttt
polyT_76	15[127]	15[145]	TAATTTGCCAGtttttttt
polyT_77	11[25]	10[43]	ttttttttGCTTGATACCGATAGTGTGAAT
polyT_78	49[18]	48[18]	ttttttttAATGTGAGCGAGTAACGCTTTCATCAACATTAtttttttt
polyT_79	70[138]	70[112]	ttttttttCACCCAAATCAAGTTTTTT
polyT+A_1	17[125]	17[142]	AGTTGCTATTtttttttt
polyT+A_2	3[131]	3[145]	TAGCAAGtttttttt
polyT+A_3	24[46]	24[22]	AACTGGCTCATTATACCtttttttt
polyT+A_4	44[145]	47[145]	ttttttttGATAATCAGAAATCCAGCCAGCTTtttttttt
polyT+A_5	19[126]	19[142]	CCTGCTCCAtttttttt
polyT+A_6	46[41]	46[25]	ACCGTAATGtttttttt

Functionality	Start	End	Sequence
polyT+A_7	64[35]	64[18]	GTTTGCGTATtttttttt
polyT+A_8	1[37]	2[25]	ACCAGGTACCGCCAtttttttt
polyT+A_9	11[127]	11[145]	GAATTGAGTTAtttttttt
polyT+A_10	71[124]	71[138]	GAACCATtttttttt
polyT+A_11	52[41]	52[22]	AGTCACGACGTTtttttttt
polyT+A_12	58[125]	61[138]	CCACCGACCACGCTGGTTTtttttttt
polyT+A_13	10[42]	10[25]	TTCTTAAACAtttttttt
polyT+A_14	70[34]	70[18]	GGAACCAGAtttttttt
polyT+A_15	50[145]	55[142]	ttttttttCCCTCAATCAATGAATTGAGGtttttttt
polyT+A_16	15[25]	16[22]	ttttttttGGTAAAATAATAGCAAGCAAAtttttttt
polyT+A_17	26[34]	26[18]	AATATTCATtttttttt
polyT+A_18	25[117]	25[142]	ATTTAGGAATACCACATTtttttttt
polyT+A_19	63[126]	63[142]	AATATCCAGtttttttt
polyT+A_20	40[42]	40[25]	GGCCGGAGACtttttttt
polyT+A_21	27[120]	27[138]	TTTTCGAGCCAtttttttt
polyT+A_22	60[42]	60[22]	AGCTCGAATTCGTtttttttt
polyT+A_23	36[118]	35[138]	TTTTCCCATAGGTCTGAGAGACTACCtttttttt
polyT+A_24	54[43]	54[22]	AGCGTAAGAATACGtttttttt
polyT+A_25	40[145]	43[145]	ttttttttTGAGAGTCTGGATGTACCCCGGTTtttttttt
polyT+A_26	34[35]	34[18]	TAGCATTAACtttttttt

Table S2. Sequences of staple strand replacements to facilitate the attachment of DNA-functionalized HRP or A488-labeled oligonucleotides. The ssDNA overhangs are marked in italics.

Functionality	Start	End	Sequence
HRP-1	14[103]	15[109]	CCAAATAAGAAACTACACTAAAAACGCTAACG*tttTGGTTGGGTGGTG*
HRP-2	32[64]	33[69]	GATAAATAATAGTATCATATGCG*tttTGGTTGGGTGGTG*
HRP-3	3[49]	7[55]	CCTCAGAAAGACACGGTGGCA*tttTGGTTGGGTGGTG*
HRP-4	62[111]	62[90]	ACGAGCACGTATAACGTGCTTT*tttTGGTTGGGTGGTG*
polyT+A488-1	17[125]	17[142]	AGTTGCTATT*TTTTTTCTCCTTTCCC*
polyT+A488-2	3[131]	3[145]	TAGCAAG*TTTTTTCTCCTTTCCC*
polyT+A488-3	24[46]	24[22]	AACTGGCTCATTATACC*TTTTTTCTCCTTTCCC*
polyT+A488-4	44[145]	47[145]	ttttttttGATAATCAGAAATCCAGCCAGCTT*TTTTTTCTCCTTTCCC*
polyT+A488-5	19[126]	19[142]	CCTGCTCCA*TTTTTTCTCCTTTCCC*
polyT+A488-6	46[41]	46[25]	ACCGTAATG*TTTTTTCTCCTTTCCC*
polyT+A488-7	64[35]	64[18]	GTTTGCGTAT*TTTTTTCTCCTTTCCC*
polyT+A488-8	1[37]	2[25]	ACCAGGTACCGCCA*TTTTTTCTCCTTTCCC*
polyT+A488-9	11[127]	11[145]	GAATTGAGTTAt*TTTTTTCTCCTTTCCC*
polyT+A488-10	71[124]	71[138]	GAACCAT*TTTTTTCTCCTTTCCC*
polyT+A488-11	52[41]	52[22]	AGTCACGACGTT*TTTTTTCTCCTTTCCC*
polyT+A488-12	58[125]	61[138]	CCACCGACCACGCTGGTTT*TTTTTTCTCCTTTCCC*
polyT+A488-13	10[42]	10[25]	TTCTTAAACA*TTTTTTCTCCTTTCCC*
polyT+A488-14	70[34]	70[18]	GGAACCAGA*TTTTTTCTCCTTTCCC*
polyT+A488-15	50[145]	55[142]	ttttttttCCCTCAATCAATGAATTGAGG*TTTTTTCTCCTTTCCC*
polyT+A488-16	15[25]	16[22]	ttttttttGGTAAAATAATAGCAAGCAAA*TTTTTTCTCCTTTCCC*
polyT+A488-17	26[34]	26[18]	AATATTCAT*TTTTTTCTCCTTTCCC*
polyT+A488-18	25[117]	25[142]	ATTTAGGAATACCACATT*TTTTTTCTCCTTTCCC*
polyT+A488-19	63[126]	63[142]	AATATCCAG*TTTTTTCTCCTTTCCC*
polyT+A488-20	40[42]	40[25]	GGCCGGAGAC*TTTTTTCTCCTTTCCC*
polyT+A488-21	27[120]	27[138]	TTTTCGAGCCA*TTTTTTCTCCTTTCCC*
polyT+A488-22	60[42]	60[22]	AGCTCGAATTCGT*TTTTTTCTCCTTTCCC*
polyT+A488-23	36[118]	35[138]	TTTTCCCATAGGTCTGAGAGACTACC*TTTTTTCTCCTTTCCC*
polyT+A488-24	54[43]	54[22]	AGCGTAAGAATACG*TTTTTTCTCCTTTCCC*
polyT+A488-25	40[145]	43[145]	ttttttttTGAGAGTCTGGATGTACCCCGGTT*TTTTTTCTCCTTTCCC*
polyT+A488-26	34[35]	34[18]	TAGCATTAAC*TTTTTTCTCCTTTCCC*
core+A488-1	46[64]	47[56]	GGCGGGGAACAAACGGTGTAGATG*TTTTTTCTCCTTTCCC*
core+A488-2	10[103]	11[103]	CAATAGCTATCTTGTAATTGAGCGCT*TTTTTTCTCCTTTCCC*

Functionality	Start	End	Sequence
core+A488-3	33[112]	34[112]	TTTTGCGCTGTAGCGTGTCTGGGCTTAG*TTTTTTCTCCTTTCCC*
core+A488-4	26[111]	26[90]	TACCGACAAGTTTTGCCAGAGG*TTTTTTCTCCTTTCCC*
core+A488-5	14[61]	11[61]	CTTTTTTCGGAACGCTGAGGACAATG*TTTTTTCTCCTTTCCC*
core+A488-6	67[63]	69[79]	CTGAAACACCCTCAGTCAGACGATTGGCCTT*TTTTTTCTCCTTTCCC*
core+A488-7	70[76]	70[54]	CCCCTTATTAGCGTTTGCCATCT*TTTTTTCTCCTTTCCC*
core+A488-8	33[70]	34[69]	TTATACATACCGACATTTTAGAATCCAAT*TTTTTTCTCCTTTCCC*
core+A488-9	61[113]	63[110]	AGCGGTGTAAAAGAGTCTGTCAAACT*TTTTTTCTCCTTTCCC*
core+A488-10	71[90]	71[113]	AAAAACCGTCTATCAGGGCGATGG*TTTTTTCTCCTTTCCC*
core+A488-11	62[48]	63[47]	GTTGCGCTAAAGCCACATACGAGCTGT*TTTTTTCTCCTTTCCC*
core+A488-12	3[106]	2[88]	AAATCACGATAGCAGCACCGTAATCAGTAG*TTTTTTCTCCTTTCCC*
core+A488-13	47[77]	43[75]	CTGCCAGGTAAACGTTAATATAGATTT*TTTTTTCTCCTTTCCC*
core+A488-14	24[102]	25[116]	TTACGGAAAGATTCATCAGTTGAG*TTTTTTCTCCTTTCCC*
core+A488-15	54[55]	55[67]	TTCTGACCAAGCTTTCTCAGGAGAAG*TTTTTTCTCCTTTCCC*
core+A488-16	47[105]	46[92]	TCGGCCTCCAGGCAAAGCGCCATTCGC*TTTTTTCTCCTTTCCC*
core+A488-17	16[90]	17[84]	TTTGACCCCCAGCGAGCGAACC*TTTTTTCTCCTTTCCC*
core+A488-18	24[68]	24[47]	CCTCGTTTACCGCGATTTTAAG*TTTTTTCTCCTTTCCC*

Table S3. Functionalized/labeled DNA oligonucleotides

Name	Sequence
HRP-oligonucleotide	/5ThioMC6-D/CACCACCCAACCA
A488-oligonucleotide	/5ATTO488N/TGGGAAAGGAGAAAAAA
A488-oligonucleotide (mismatched)	TATTGGGAATAAGTTTATTACCAGC/3ATTO488N/

Note S10: Estimation of DNA origami concentration

The concentration of different NR variants was estimated using Lambert-Beer's law, with the variant-specific extinction co-effienct being estimated from the number of hybridized and non-hybridized nucleotides (5).

Table S4. Extinction coefficient of different NR variants

DNA origami variant	Extinction coefficient (M^{-1} cm^{-1})
NR/NH	1.13×10^8
NR-E (w/o A488)	1.15×10^8
NA (≙ NR-E+A488)	1.05×10^8
NR-F	1.16×10^8

Note S11: Preparation of virus CPs

Isolation of CCMV CPs

Native CCMV particles were cultured in cowpea plants. Briefly, plant material was harvested after seven to ten days after initial infection with CCMV (infected leaves or a suspension of purified CCMV particles for inoculation of ten-day old plants), followed by homogenization in 0.2 M sodium acetate buffer, pH 4.8, supplemented with 0.01 M ascorbic acid and 0.01 mM disodium EDTA. The solution was pressed through a cheesecloth and the leaf tissue pelleted after an 1 h incubation at 4 °C in a centrifugation step using 10,000 rpm, 4 °C for 10 min. The supernatant containing the CCMV was collected and 10 % (w/v) PEG (MW = 6,000 g mol^{-1}) were dissolved in it in order to precipitate the CCMV by centrifuging at 10,000 rpm, 4 °C for 15 min. Subsequently, the pellet was resuspended in cold virus buffer containing 0.1 M sodium acetate, pH 5.0, supplemented with 1 mM sodium azide and 1 mM EDTA. Further purification was performed by pelleting undissolvable material (10,000 rpm, 4 °C, 10 min) and cesium chloride (37.5 % (w/v)) based density gradient centrifugation (40,000 rpm, 10 °C, $\leq$ 16 h) by collecting the brownish, virus containing fraction, which was then dialysed (3 $\times$ 3 h, 4 °C) against virus buffer. (6)

Subsequently, the native virus particles were disassembled by ON dialysis against 50 mM Tris-HCl, 500 mM $CaCl_2$, 1 mM dithiothreitol (DTT), pH 7.5 using Slize-A-Lyzer Mini Dialysis cups (3.5 kDa MWCO, Thermo Scientific), followed by a centrifugation step at 21,000 g, 4 °C for at least 6 h to pellet the RNA. The supernatant was then dialysed ON against 'clean buffer' containing 50 mM Tris-HCl, 150 mM NaCl, 1 mM DTT. Using a molar extinction coefficient of 23,590 M^{-1} cm^{-1},

the concentration of the CPs was determined based on their absorbance at 280 nm (BioTek Eon Microplate Spectrophotometer, 2 μL sample, Take3 plate). (7)

Preparation of MPyV capsomers

Wildtype VP1 MPyV proteins were prepared as described previously (7) by recombinant expression in *E. coli* Rosetta (D3) pLysS cells (Novagen). Briefly, 5 mL of a starting culture (terrific broth (TB) media consisting of 12 g L^{-1} tryptone, 24 g L^{-1} yeast extract, 0.4 % (v/v) glycerol, 12.24 g L^{-1} K_2HPO_4, 2.31 g L^{-1} KH_2PO_4; 34 mg L^{-1} chloramphenicol and 100 mg L^{-1} ampicillin were used as antibiotics) was inoculated with a single colony and left to grow ON at 30 °C and 180 rpm. The preculture was diluted into 500 mL TB media, supplemented with 34 mg L^{-1} chloramphenicol and 100 mg L^{-1} ampicillin, and the growth temperature increased to 37 °C. Once an optical density at 600 nm (OD_{600}) of 0.5–0.6 was reached, the cells were cooled down using an ice bath. Protein expression was induced with 0.3 mM isopropyl β-D-thiogalactopyranoside (IPTG). After 16 h at 26 °C, the cells were harvested in a centrifugation step (15 min, 4,000*g*, 4 °C) and stored until further use at -20 °C.

For the purification of the VP1, the cell pellets were first resuspended in approx. 40 mL of 'MPyV clean buffer', which contained 40 mM Tris, pH 8.0, 200 mM NaCl, 1 mM EDTA, 5 mM DTT, and 5 % (v/v) glycerol. After a sonication step, which consisted of 3×20 s bursts (20 % output, 1 min pause between pulses) and was performed on ice, the lysate was centrifuged for 25 min at 25,000*g*, 4 °C. The protein containing supernatant is further purified using affinity and size exclusion chromatography (ÄKTA Pure, Cytiva/NGC discover, Bio-Rad) by utilizing a glutathione-S-transferase (GST) tag which is linked through a thrombin cleavage site and located at the *N*-terminus of VP1. To this end, the filtered supernatant (0.45 μm syringe filter, Merck Millipore) was loaded onto an equilibrated GST Trap FF column (5 mL, GE Healthcare, 0.5 mL min^{-1} flow rate). The column was first washed with MPyV clean buffer, followed by manually loading 5 mL of MPyV clean buffer supplemented with thrombin (50–100 units) and sealing the column. After a 16 h incubation period at 4 °C to allow thrombin to free the VP1 from the column, VP1 was eluted in MPyV clean buffer. Possible aggregates were removed by passing the elute through an equilibrated (with MPyV clean buffer) size exclusion column (Superdex 200 10/300 GL column, GE Healthcare). In order to *in vitro* reassemble VP1 into VLPs dialysis against 'assembly buffer' (0.5 M $(NH_4)_2SO_4$, 20 mM Tris, pH 7.4, 5 % (v/v) glycerol, and 1 mM $CaCl_2$) was performed ON at 4 °C (Slize-A-Lyzer Mini Dialysis cups, 3.5 kDa MWCO, Thermo Scientific).

The concentration of purified VP1 was estimated using Lambert-Beer's law (monomer extinction coefficient 57,870 M^{-1} cm^{-1}) by measuring their absorbance at 280 nm (BioTek Eon Microplate Spectrophotometer, Take3 plate, 2 μL sample volume).

Note S12: Preparation of p-anti-HER2

The p-anti-HER2 was prepared as described previously (8). Briefly, the anti-HER2 was recombinantly expressed in RV308 strain (*E. coli*). To this end, a starting culture (16 mL, lysogeny broth medium) was grown over night at 37 °C, 220 rpm. The medium was supplemented with 1 % (v/v) glucose and 400 μg mL^{-1} ampicillin. Subsequently, the starting culture was diluted (2 % (v/v) to 400 mL of main culture (terrific broth medium, supplemented with 100 μg mL^{-1} ampicillin) and the bacteria grown at 37 °C, 180 rpm until an OD_{600} of 4.0–5.0 was reached. IPTG (final concentration 1 mM) was added to induce the protein expression (16–20 h, 30 °C, 180 rpm), after which the cells were harvested (13,700*g*, 15 min).

The anti-HER2 has an engineered His-Tag at its C-terminus, which facilitated the purification from the medium. His-beads (2.5 mL resin for 170 mL medium; HisPur Ni-NTA resin, ThermoScientific, prepared according to manufacturer's instructions) were incubated with 170 mL of medium and equal volume of equilibration buffer (20 mM sodium phosphate, pH 7.4, supplemented with 300 mM NaCl and 10 mM imidazole) for 1 h on an end-to-end shaker (RT). Washing (20 mM sodium phosphate, pH 7.4, supplemented with 300 mM NaCl and 25 mM imidazole) and elution (20 mM sodium phosphate, pH 7.4, supplemented with 300 mM NaCl and 250 mM imidazole) were performed by sedimenting the His-beads by centrifugation at 700*g*, 4 °C for 2 min. For the washing steps (total 6×) two resin-bed volumes where used, whereas the proteins were eluted (total 5×) in one resin-bed volume per repetition. The eluted fractions were pooled and filtered (0.45 μm), followed by an upconcentration step using spin-filtration (10 kDa MWCO PES, Vivaspin 20, Sartorius, 20 mL) for 10 min at 3,200*g*. To ensure a reduced free Cys-residue at the C-terminus, the protein solution was treated with DTT (final concentration 2 mM) for 30 min at 37 °C. Subsequently, the solution was loaded onto a desalting column (HiTrap Desalting, 2×5 mL bead volume, Cytvia) and eluted in 1×PBS, pH 6, supplemented with 1 mM EDTA. The collected protein containing fractions were pooled and their concentration determined by measuring the absorbance at 280 nm (extinction coefficient 50,100 M^{-1} cm^{-1}).

For the conjugation reaction of anti-HER2 with the artificial binding domain, which was carried out in 1× PBS pH 7, a second generation Newcome-type dendrimer (G2, +27, Figure S15) was mixed with the protein at 2× molar excess. After an incubation at RT for 2 h on an end-to-end shaker, the reaction mixture was transferred to 4 °C (∼40 h) and subsequently loaded onto a cation ion exchange column (HiTrap Heparin column, 5 mL) to remove unreacted components. anti-HER2-G2 was eluted using a gradient (20 mM HEPES buffer, pH 7, supplemented with 20 mM EDTA and 0–2 M NaCl) and upconcentrated (10 kDa

MWCO PES, 10 min, 3,200*g*). The salt was removed by dialysis against 10 mM HEPES, pH 7.0 (10 kDa MWCO dialysis cups).

Bibliography

1. Välimäki, S.; Khakalo, A.; Ora, A.; Johansson, L.-S.; Rojas, O. J.; Kostiainen, M. A. Effect of PEG–PDMAEMA Block Copolymer Architecture on Polyelectrolyte Complex Formation with Heparin. *Biomacromolecules* **2016**, *17*, 2891–2900, https://doi.org/10.1021/acs.biomac.6b00699.
2. Ijäs, H.; Hakaste, I.; Shen, B.; Kostiainen, M. A.; Linko, V. Reconfigurable DNA Origami Nanocapsule for pH-Controlled Encapsulation and Display of Cargo. *ACS Nano* **2019**, *13*, 5959–5967, https://doi.org/10.1021/acsnano.9b01857.
3. Julin, S.; Nonappa; Shen, B.; Linko, V.; Kostiainen, M. A. DNA-Origami-Templated Growth of Multilamellar Lipid Assemblies. *Angew. Chem. Int. Ed.* **2021**, *60*, 827–833, https://doi.org/10.1002/anie.202006044.
4. Kostiainen, M. A.; Szilvay, G. R.; Lehtinen, J.; Smith, D. K.; Linder, M. B.; Urtti, A.; Ikkala, O. Precisely Defined Protein–Polymer Conjugates: Construction of Synthetic DNA Binding Domains on Proteins by Using Multivalent Dendrons. *ACS Nano* **2007**, *1*, 103–113, https://doi.org/10.1021/nn700053y.
5. Hung, A. M.; Micheel, C. M.; Bozano, L. D.; Osterbur, L. W.; Wallraff, G. M.; Cha, J. N. Large-Area Spatially Ordered Arrays of Gold Nanoparticles Directed by Lithographically Confined DNA Origami. *Nat. Nanotechnol.* **2010**, *5*, 121–126, https://doi.org/10.1038/nnano.2009.450.
6. Comellas Aragones, M. *The Cowpea Chlorotic Mottle Virus As a Building Block in Nanotechnology*; ISBN: 9789090249339, Disseration, RU Radboud Universiteit Nijmegen, 2010; https://hdl.handle.net/2066/74710.
7. Seitz, I.; Saarinen, S.; Kumpula, E.-P.; McNeale, D.; Anaya-Plaza, E.; Lampinen, V.; Hytönen, V. P.; Sainsbury, F.; Cornelissen, J. J.; Linko, V.; Huiskonen, J. T.; Kostiainen, M. A. DNA-Origami-Directed Virus Capsid Polymorphism. *Nat. Nanotechnol.* **2023**, *18*, 1205–1212, https://doi.org/10.1038/s41565-023-01443-x.
8. Seitz, I.; Ijäs, H.; Linko, V.; Kostiainen, M. A. Optically Responsive Protein Coating of DNA Origami for Triggered Antigen Targeting. *ACS Appl. Mater. Interfaces* **2022**, *14*, 38515–38524, https://doi.org/10.1021/acsami.2c10058.
